# Integration of Single-Cell and Bulk RNA Sequencing Data to Identify Lactylation-Related Gene Signatures in Hepatic Ischemia–Reperfusion Injury Using Machine Learning Algorithms

**DOI:** 10.3390/ijms27177965

**Published:** 2026-09-07

**Authors:** Shilei Jing, Zhijun Zhu

**Affiliations:** 1 Liver Transplantation Center, National Clinical Research Center for Digestive Diseases, Beijing Friendship Hospital, Capital Medical University, Beijing 100050, China; 21718127@zju.edu.cn; 2 Laboratory for Clinical Medicine, Capital Medical University, Beijing 100050, China; 3 State Key Laboratory of Digestive Health, Beijing 100050, China; 4 Beijing Key Laboratory of Organ Cultivation and Organ Protection in Transplantation, Beijing Friendship Hospital, Capital Medical University, Beijing 100050, China; 5 Clinical Center for Pediatric Liver Transplantation, Capital Medical University, Beijing 100050, China

**Keywords:** lactylation-related genes, hepatic ischemia–reperfusion injury (HIRI), multi-omics, machine learning algorithms

## Abstract

Hepatic ischemia–reperfusion injury (HIRI) is not only a common complication of liver transplantation and major hepatic surgery but also a critical determinant of postoperative prognosis. Lactate metabolic reprogramming has been observed in HIRI, yet the role of lactate and its related lactylation in the pathogenesis of HIRI remains unclear. To address this, we integrated single-cell and bulk RNA-seq data with multiple bioinformatic approaches. Five single-cell gene set activity scoring methods (AUCell, UCell, singscore, ssGSEA, and AddModuleScore) were applied to evaluate lactylation activity across cell types, followed by differentially expressed gene (DEG) analysis and high-dimensional Weighted Correlation Network Analysis (hdWGCNA) to identify lactylation-associated genes. Five machine learning algorithms (Random Forest, Boruta, LASSO, GBM, and Decision Tree) were used to screen optimal feature genes, with SHAP analysis further explaining their importance. Bulk RNA sequencing data from the Gene Expression Omnibus (GEO) database were used for validation. Furthermore, NR4A3-related inhibitors were screened using the ChEMBL online tool and assessed by docking and molecular dynamic simulation. We observed significant heterogeneity in lactate metabolism activity across cell types in hepatic ischemia–reperfusion injury (HIRI), with higher activity levels observed for hepatocytes and mononuclear phagocytes. The integration of SHAP and machine learning identified PFKFB3, ZYX, and NR4A3 as closely associated with high lactylation after HIRI, and cross-analysis with bulk RNA data confirmed their consistent upregulation. Candidate gene expression was experimentally validated in a murine liver IRI model through Western blotting and RT-qPCR. Although lactylation has been previously reported in HIRI, this study’s unique contribution is to reveal the cell-type heterogeneity of lactylation-related gene expression at the single-cell level through multi-omics integration and machine learning. The identification of NR4A3, PFKFB3, and ZYX as lactylation-associated regulators proposes novel therapeutic targets for improving graft survival in liver transplantation.

## 1. Introduction

Hepatic ischemia–reperfusion injury (HIRI) remains a critical clinical challenge and is typically encountered in hepatectomy, liver transplantation, and hemorrhagic shock [1]. The condition can lead to severe liver dysfunction or even multiple organ failure, significantly impacting the long-term prognosis of patients [2]. In fact, HIRI has been linked to approximately 10% of early acute rejection cases following liver transplantation and also predisposes the recipient liver to an increased risk of tumor recurrence, fibrosis, and viral hepatitis [3,4]. The pathogenesis underlying HIRI includes two distinct stages, ischemic injury and reperfusion insult, which are characterized by a complex inflammatory response that engages multiple key factors. These key factors include aerobic metabolism disturbances, oxidative stress, macrophage polarization, apoptosis, sodium pump inactivation, mitochondrial dysfunction, calcium overload, DNA damage, immune activation, and inflammatory mediators [5,6,7]. Despite extensive research into the mechanisms underlying HIRI, the molecular events governing this process have not been fully elucidated, underscoring the urgent need for innovative therapeutic strategies to mitigate this injury.

Lactylation is a novel post-translational modification (PTM) that involves the covalent attachment of lactate molecules to lysine residues, thereby altering their activity and function [8]. This process is mediated primarily by lactyltransferases and delactylases. It has emerged as a critical regulatory mechanism in various cellular processes, including energy regulation, modulation of inflammation and immune responses, and regulation of metabolic reprogramming [9,10,11]. Current studies indicate that lactylation acts as a core metabolic–epigenetic switch during ischemia–reperfusion injury (IRI) in various organ systems. During cerebral ischemia–reperfusion, astrocytic low-density lipoprotein receptor-related protein-1 (LRP1) suppresses ARF1 lactylation, thereby promoting mitochondrial transfer from astrocytes to neurons and exerting a neuroprotective effect [12]. These findings suggest that lactylation may play a critical role in ischemia–reperfusion injury across different organ systems. Recent studies have revealed that lactate-induced mitochondrial calcium uptake 3 aggravates myocardial ischemia–reperfusion injury by promoting neutrophil extracellular trap formation [13]. In the context of HIRI, lactylation of mitochondrial phosphoenolpyruvate carboxykinase 2 (PCK2) has been shown to promote mitochondrial fatty-acid synthesis, thereby exacerbating ferroptosis [14]. Meanwhile, Du et al. reported that the overexpression of HSPA12A reduces glycolysis-generated lactate, which in turn decreases HMGB1 lactylation and its secretion from hepatocytes, ultimately inhibiting macrophage chemotaxis and the subsequent inflammatory cascade, thus conferring protection against ischemia–reperfusion injury [15]. Despite these advances, the specific role and mechanisms of lactylation-mediated regulation in HIRI remain largely unexplored, highlighting the need for a more systematic investigation of its potential impact.

With the continuous advancement of sequencing technology, single-cell RNA sequencing (scRNA-seq) has become a pivotal tool in the biomedical field, providing an approach to analyzing cellular heterogeneity and intercellular communication [16]. The integration of multiple machine learning algorithms with the SHAP analysis approach has further enhanced the identification of potential biomarkers [17,18]. In this study, we were the first to uncover the cellular heterogeneity of lactylation metabolism in hepatic ischemia–reperfusion injury by integrating scRNA-seq and bulk RNA-seq data. This endeavor aims at fostering the systematic identification and more comprehensive understanding of lactylation-related gene signatures in hepatic ischemia–reperfusion injury, which may serve as new therapeutic targets and provide critical insights for future mechanism research.

## 2. Results

### 2.1. Analyzing the Complexity of Lactylation-Related Genes (LRGs) in HIRI Using scRNA-Seq

**Figure 1:**
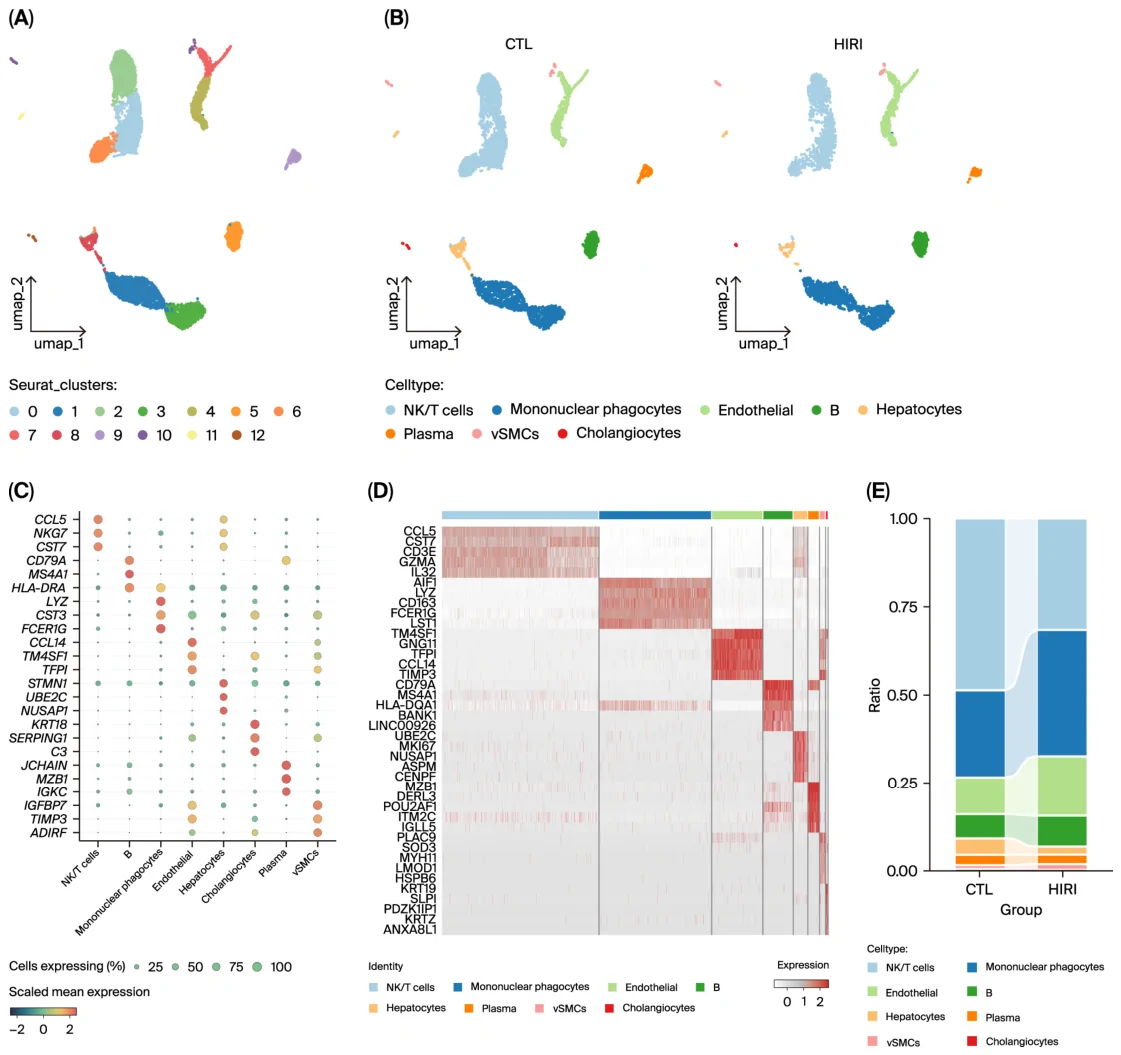

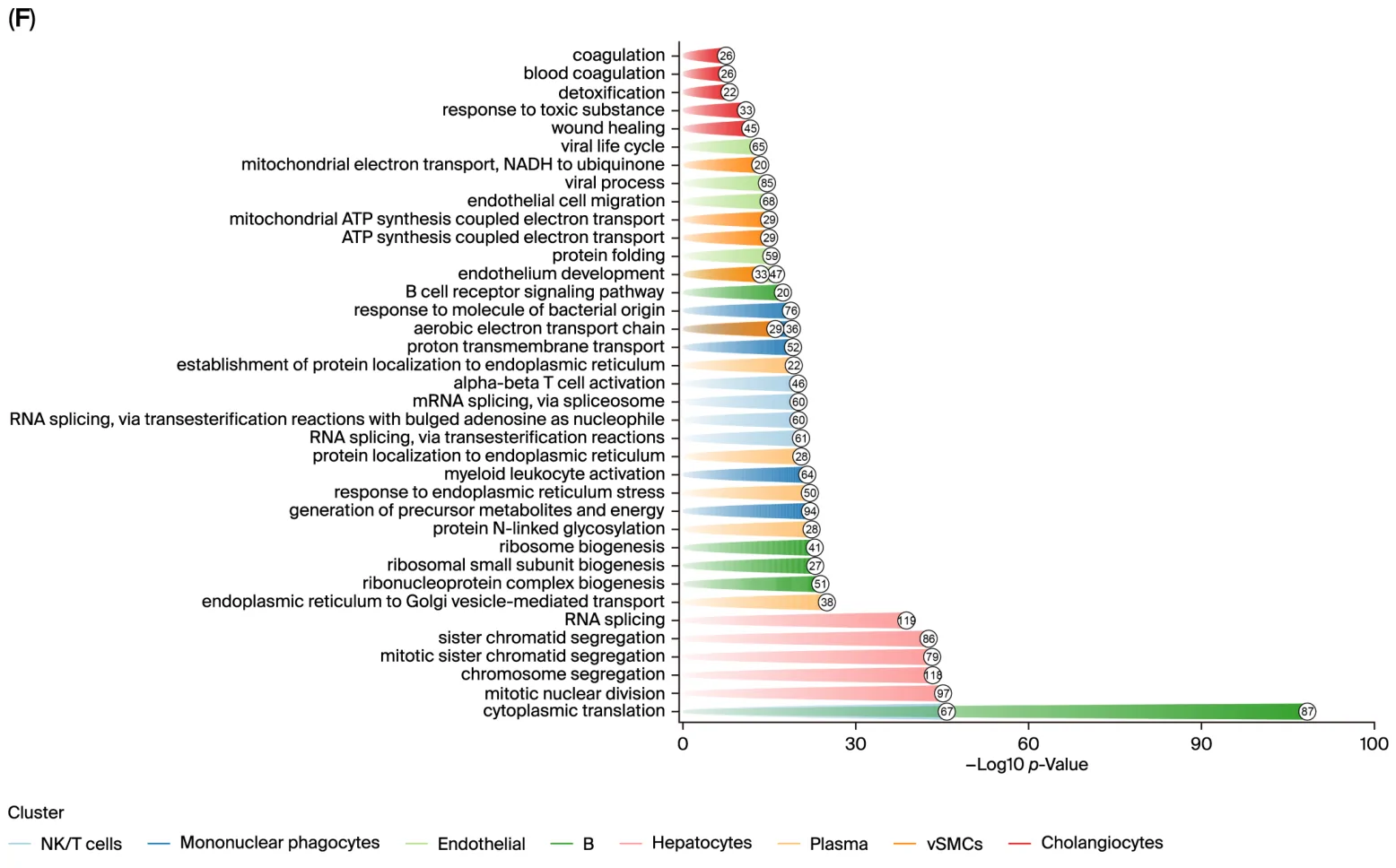
Single-cell landscape in HIRI. (**A**) The results of UMAP indicated that all cells were meticulously classified into 13 clusters. (**B**) The UMAP of cells from all scRNA-seq samples, colored by cell-type annotation. (**C**,**D**) Dot plot and heatmap of marker genes for each cell type. (**E**) Bar diagram of the proportion of each cell type in the two groups. (**F**) The relevant pathways enriched by GO analysis based on the eight distinct cell types.

**Figure 2:**
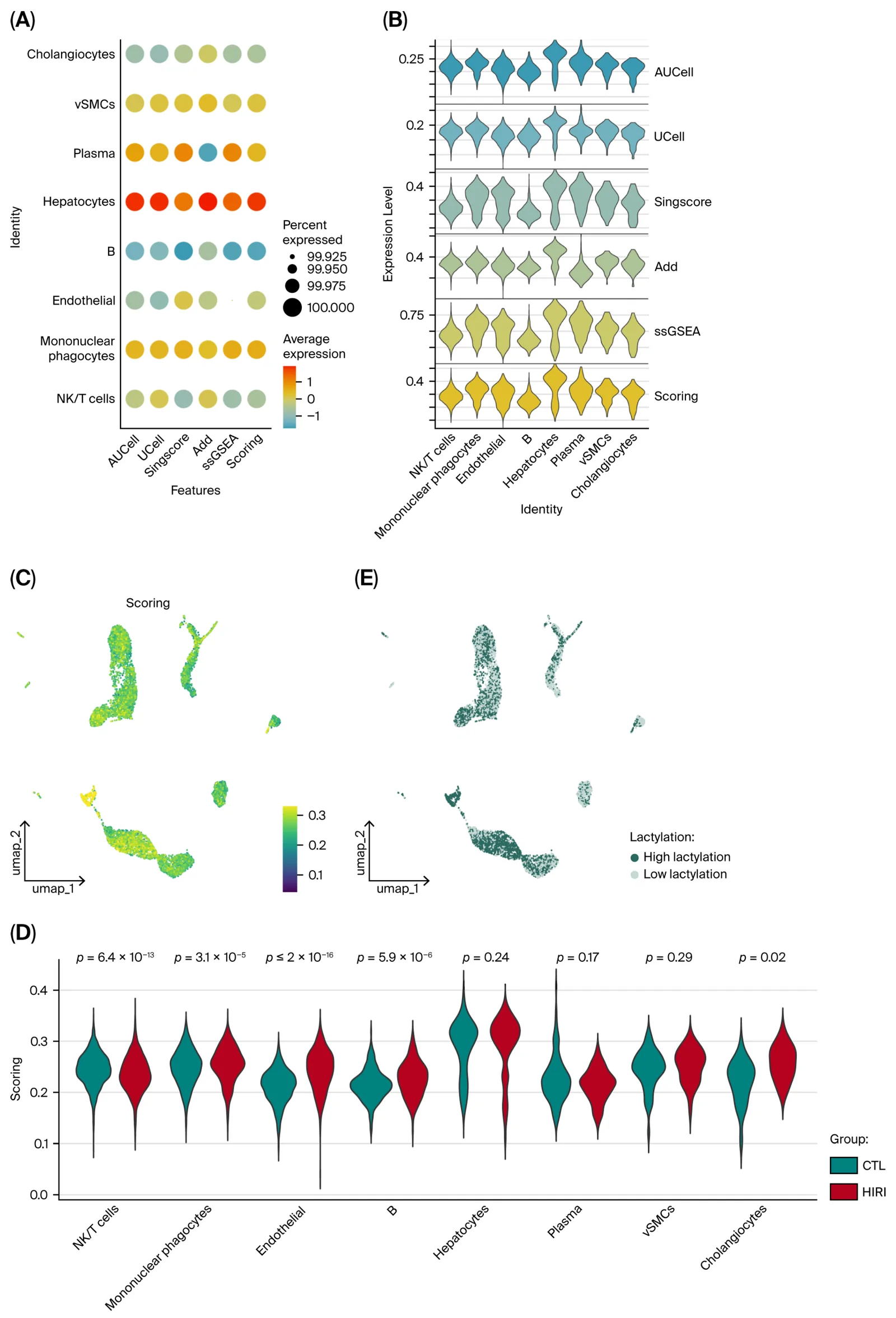
Analysis of single-cell lactylation-related genes (LRGs) activity in HIRI. (**A**,**B**) The results of the AUCell, Ucell, singscore, ssgsea, and AddModuleScore algorithms revealed lactylation-related genes (LRGs) activity via dot plots (**A**) and violin plots (**B**). (**C**) Density plot of scores for the lactylation-related gene (LRG) set. (**D**) Differences in the lactylation-related gene (LRG) scores for each cell type in CTL and HIRI samples. (**E**) According to the median total lactylation-related gene (LRG) score, all cells were classified as high-lactylation or low-lactylation cells.

Using the Seurat pipeline, we classified all cells into 13 clusters (Figure 1A), which were then assigned to eight distinct cell types based on classical marker genes, namely, NK/T cells, mononuclear phagocytes, endothelial cells, B cells, hepatocytes, plasma cells, vascular smooth muscle cells (vSMCs), and cholangiocytes (Figure 1B). The accuracy of this annotation was supported by bubble plots and heatmaps of the labeled genes (Figure 1C,D). Notably, HIRI tissues exhibited a higher proportion of mononuclear phagocytes and endothelial cells, whereas the fraction of NK/T cells decreased (Figure 1E). To further characterize these cell populations, we performed GO enrichment analysis for each subset, providing a comprehensive view of their functional features (Figure 1F). To evaluate the activity of lactylation-related genes (LRGs) at the single-cell level following HIRI, we used AUCell, Ucell, Singscore, ssGSEA, and AddModuleScore algorithms to calculate the LRG activity for each cell. These analyses reveal marked heterogeneity across cell populations after HIRI (Figure 2A–C), with hepatocytes exhibiting higher activity, whereas cholangiocytes and B cells showed lower levels. Violin plots further illustrate the distribution of LRG activity across cell types in control and HIRI tissues. We observed that endothelial cells, NK/T cells, and mononuclear phagocytes exhibited significant increases in lactylation activity scores following HIRI, whereas hepatocytes did not show a significant change (*p* = 0.24) (Figure 2D). Finally, based on the median LRG activity score, we stratified all cells into high- and low-lactylation groups (Figure 2E).

### 2.2. Identification of Key Genes by hdWGCNA

**Figure 3:**
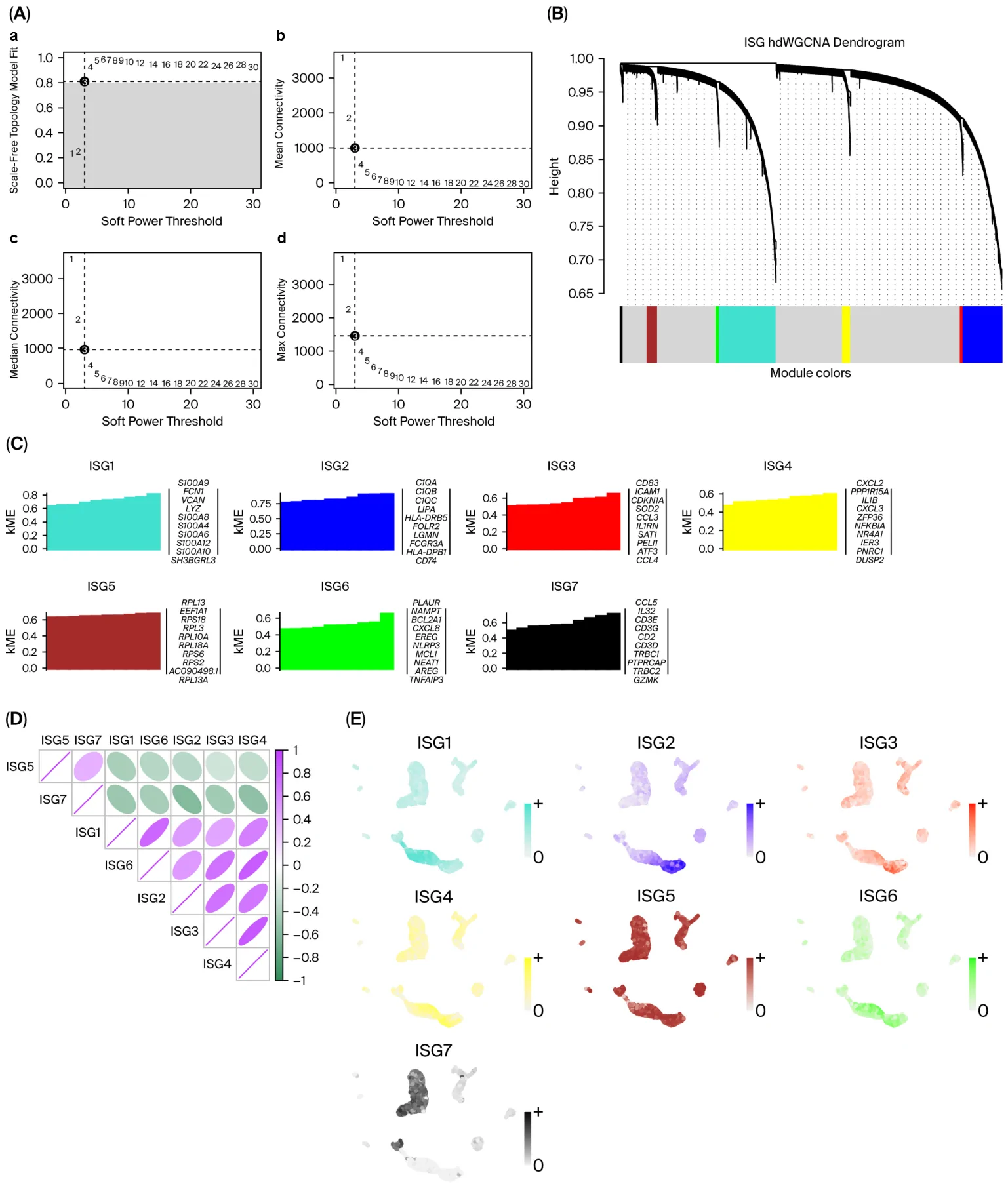

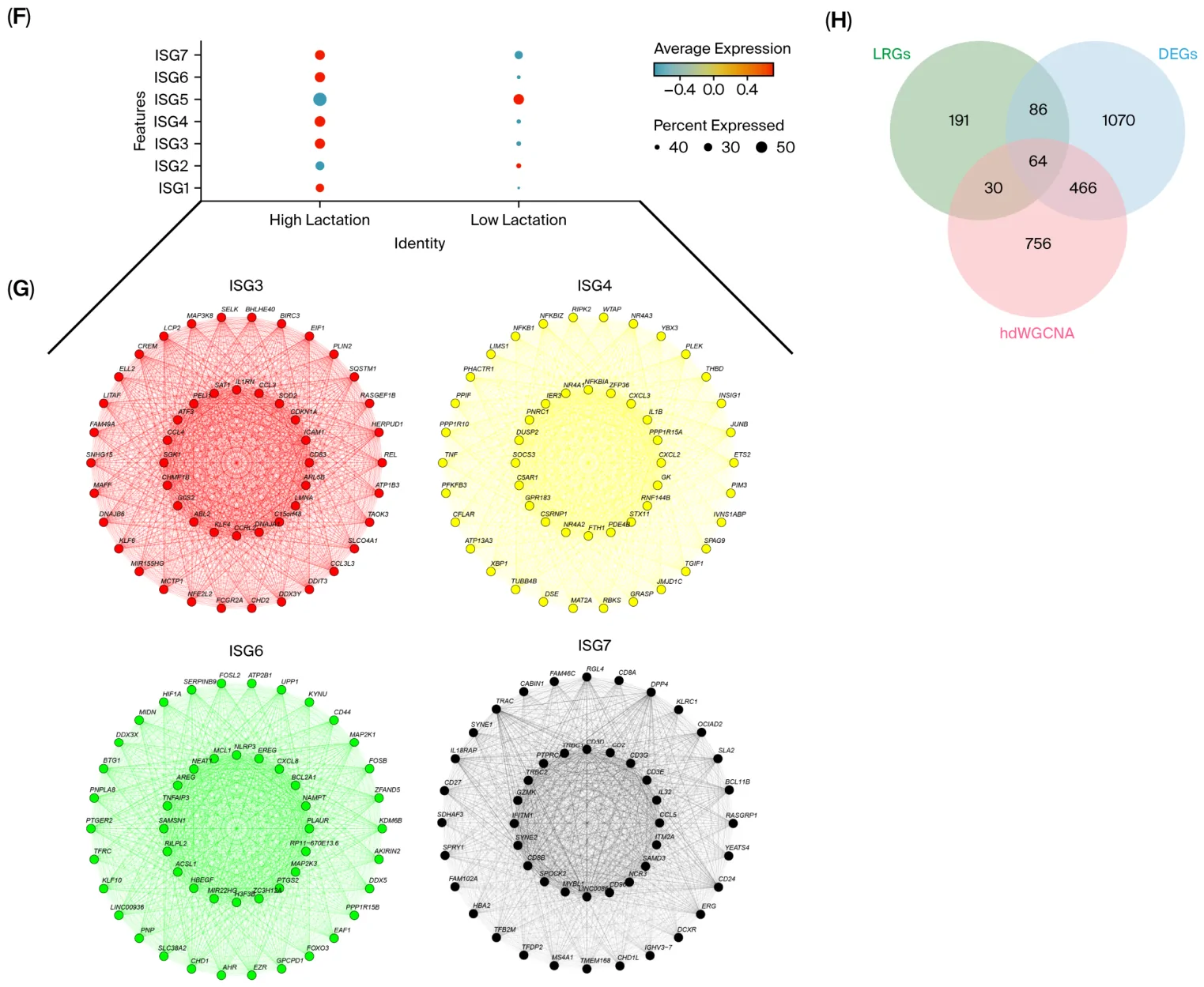
Identification of the crucial modules related to lactylation-related genes (LRGs) by hdWGCNA. (**A**) Network topology analysis across different soft power thresholds, showing scale-free fit (Panel a), mean connectivity (Panel b), median connectivity (Panel c), and maximum connectivity (Panel d). (**B**) A total of seven co-expression modules were identified. (**C**) Top hub genes within each module, ranked by kME values. (**D**) Pairwise correlation analysis among the seven modules. (**E**) UMAP plots depicting the expression density of each module. (**F**) Bubble plot showing module activity scores across different cell types. (**G**) Network visualization of the four hub modules. (**H**) Venn diagram of lactylation-related genes (LRGs), hdWGCNA, and DEG analysis.

To further pinpoint core genes linked to lactylation-related gene (LRG) activity, we constructed transcriptomic co-expression networks using high-dimensional WGCNA (hdWGCNA). Setting the soft threshold at 6, we generated seven functional co-expression modules (Figure 3A,B). Within each module, we ranked genes by their kME values and extracted the top 10 hub genes (Figure 3C; Table S2). We then assessed the pairwise correlations among these modules (Figure 3D). The examination of module expression across different cell types revealed marked enrichment of modules 3, 4, 6, and 7 in specific cell populations (Figure 3E,F). To gain insight into the functional roles of the genes within these four modules, we extracted a total of 1316 genes from the corresponding module assignments (Figure 3G; Table S3). Through DEG analysis, we identified 1686 upregulated genes between high- and low-lactylation activity groups (Table S3). Ultimately, we identified a hub gene set of 64 genes using Venn diagram intersection (Figure 3H; Table S3).

### 2.3. Identification of Core Lactylation-Promoting Genes with Multiple Machine Learning Algorithms

**Figure 4:**
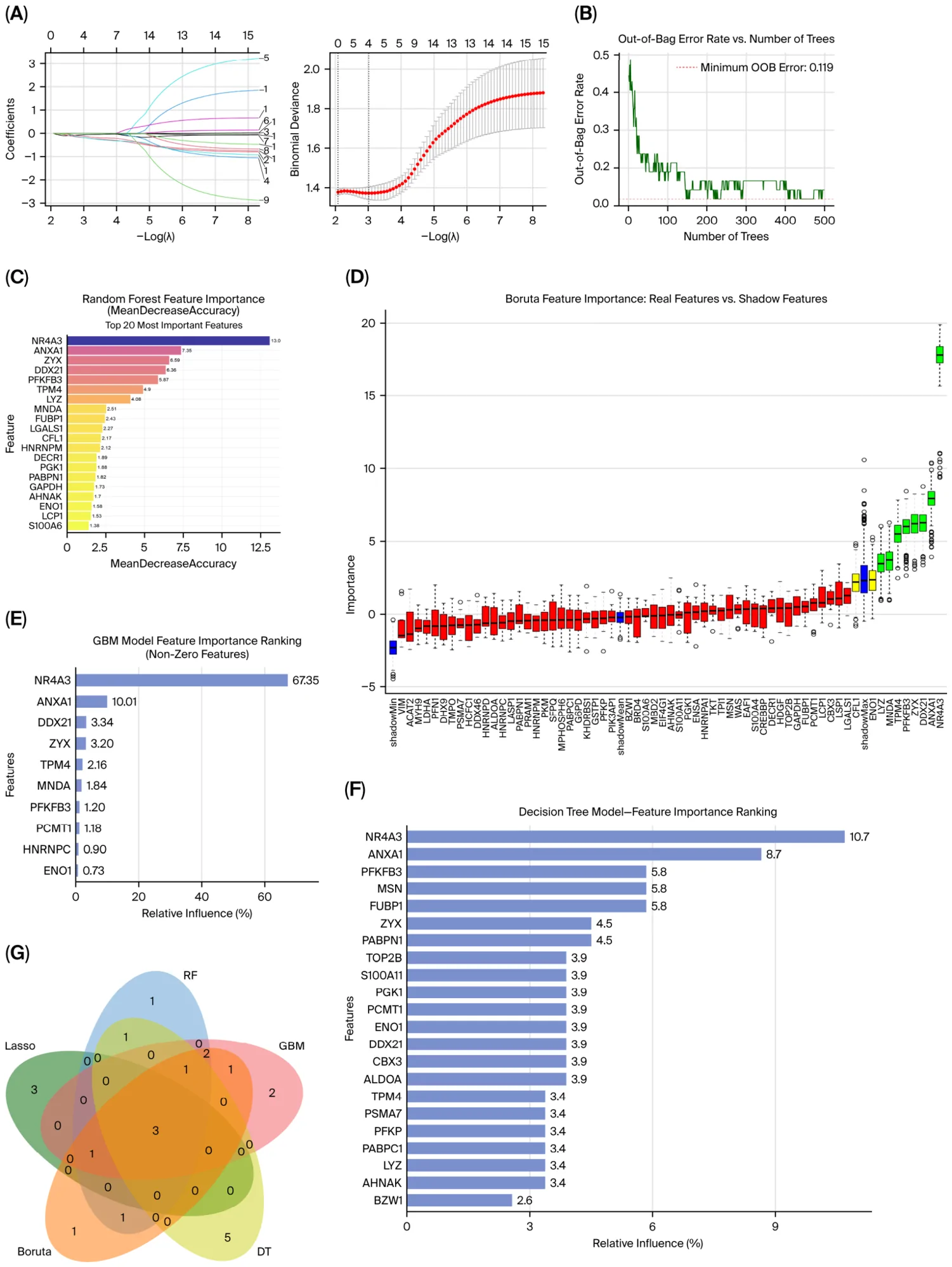
Results of the Venn diagram of lactylation-related genes (LRGs), hdWGCNA, and DEG analysis. Multiple machine learning algorithms were integrated to identify optimal feature genes. (**A**) LASSO regression for feature gene selection. Left panel: LASSO coefficient profiles of candidate feature genes; each colored line represents the coefficient trajectory of an individual gene as −Log(λ) changes. Right panel: binomial deviance plot for determining the optimal λ value. The two vertical dashed lines indicate the λ value corresponding to the minimum binomial deviance and the λ value of one standard error (1-SE), respectively. (**B**) Out-of-bag (OOB) error rate of the Random Forest model with increasing number of decision trees. The green curve shows the OOB error rate corresponding to different tree counts. The black dashed horizontal line marks the minimum OOB error (0.119) achieved in this model. (**C**) Relative importance ranking of feature genes derived from Random Forest. (**D**) Iterative trajectory of the Boruta algorithm.Boxplots show the distribution of importance (z-score) for each feature across Boruta iterations; dots represent individual importance scores from each iteration. Colors indicate shadow features (blue), confirmed important features (green), tentative (yellow) and rejected (red) features. (**E**) Feature contributions identified by the GBM algorithm. (**F**) Variable importance assessed by the Decision Tree (DT) algorithm. (**G**) Venn diagram revealing three feature genes commonly selected by all five algorithms.

Five machine learning algorithms were employed to identify core lactylation genes for HIRI. The LASSO algorithm identified seven key genes (Figure 4A; Table S4). For the Random Forest model, Figure 4B shows the out-of-bag error curve demonstrating model convergence. Ten key feature genes were obtained according to feature importance metrics (Figure 4C; Table S5). We also used the Boruta algorithm to filter out 10 feature genes after 500 iterations (Figure 4D; Table S6). For the GBM algorithm, we determined 10 top feature genes (Figure 4E; Table S7). Additionally, 10 genes were pinpointed using Decision Tree (Figure 4F; Table S8). Finally, by integrating the feature gene sets from the five machine learning algorithms above, three overlapping optimal feature genes were identified: NR4A3, PFKFB3, and ZYX (Figure 4G; Table S9). These three genes maintained stable predictive performance in both internal and external validation datasets, further verifying their robustness in HIRI.

### 2.4. Results of SHAP Analysis and Validation at the Bulk Level

**Figure 5:**
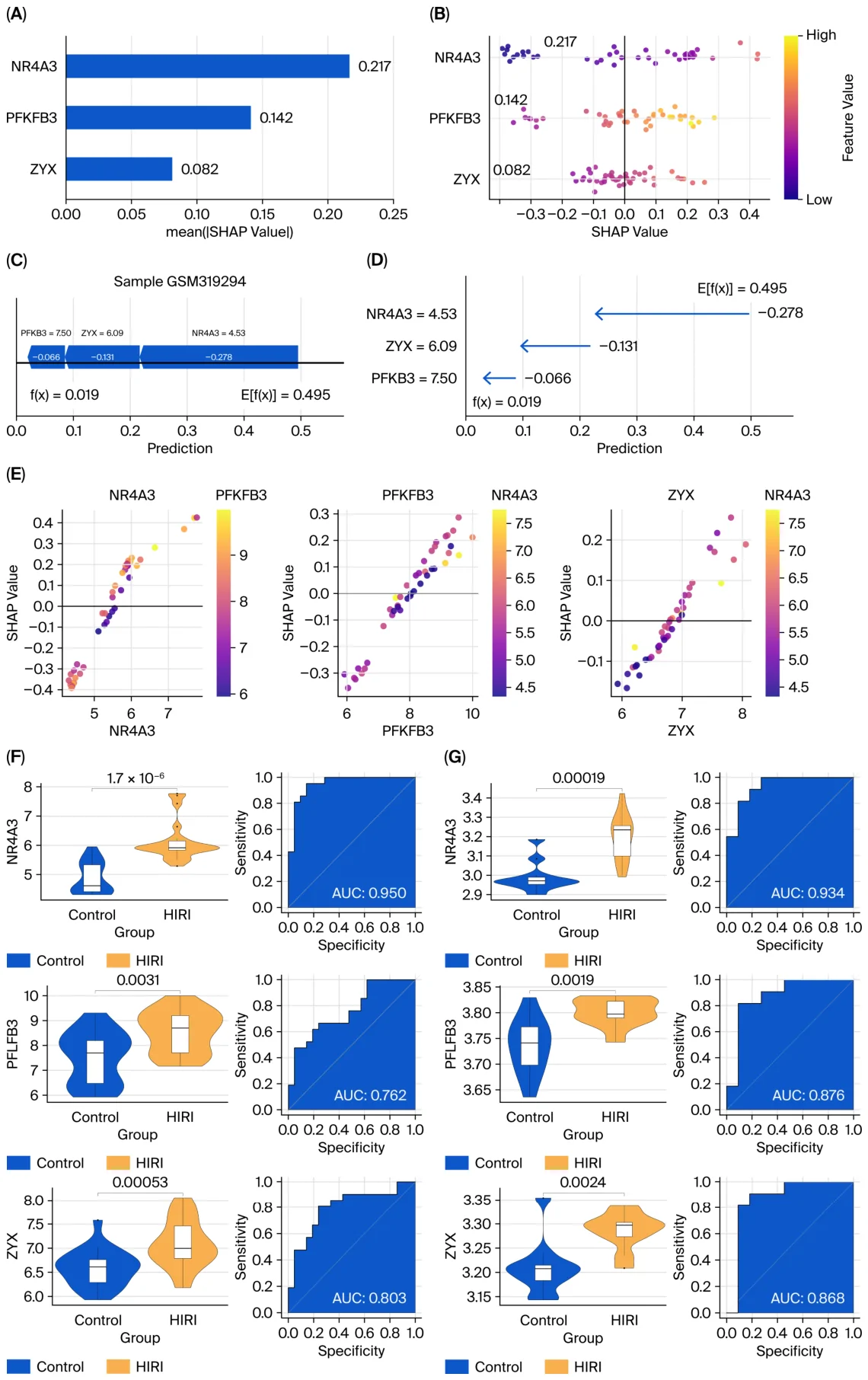
Results of SHAP analysis and validation at the bulk level. (**A**) Feature ranking by mean absolute SHAP values. (**B**) The bee colony plot, with the vertical axis representing gene names and the horizontal axis representing SHAP values, allowed us to obtain the mean SHAP value for each gene. (**C**,**D**) Decision plots illustrating cumulative contributions of individual features to risk scores. (**E**) SHAP dependence plots highlighting interactions among feature genes. (**F**) Box plot and ROC score of the characteristic genes present in the training dataset (GSE12720). (**G**) Box plot and ROC score of the characteristic genes present in the validation dataset (GSE112713).

To enhance model interpretability, we applied SHAP analysis to quantify the contribution of each feature gene. Based on the mean absolute SHAP values, NR4A3 emerged as the most influential predictor (mean SHAP = 0.217), followed by PFKFB3 (0.142) and ZYX (0.082) (Figure 5A). The swarm plot illustrates the distribution and direction of the contribution of each characterized gene to the model prediction (Figure 5B). The waterfall and force plots further demonstrate how individual features collectively shape the risk predictions (Figure 5C,D). SHAP dependence plots reveal that PFKFB3 and ZYX interact functionally with NR4A3 (Figure 5E).

To assess the diagnostic accuracy of the three candidate genes, we examined their expression in bulk RNA-seq datasets. In the GSE12720 training cohort, NR4A3, PFKFB3, and ZYX were significantly upregulated in the HIRI samples, with AUC values of 0.950, 0.762, and 0.803, respectively (Figure 5F). These findings were further validated in the independent GSE112713 cohort, where all three genes exhibited significant upregulation as well as favorable diagnostic performance, yielding AUC values of 0.934, 0.876, and 0.868 for NR4A3, PFKFB3, and ZYX, respectively (Figure 5G).

### 2.5. Validation of the Optimal Feature Genes at the Single-Cell Level

**Figure 6:**
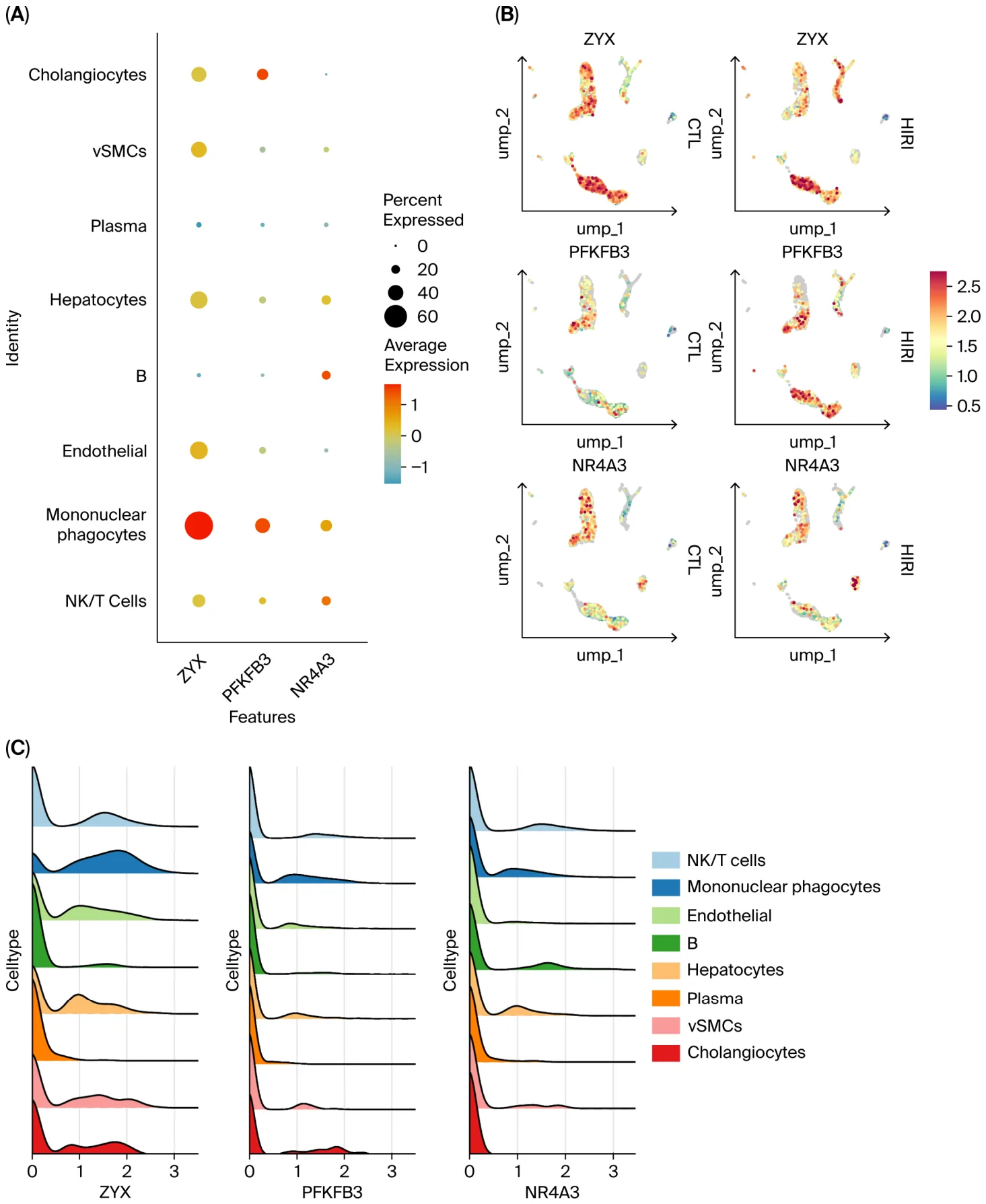

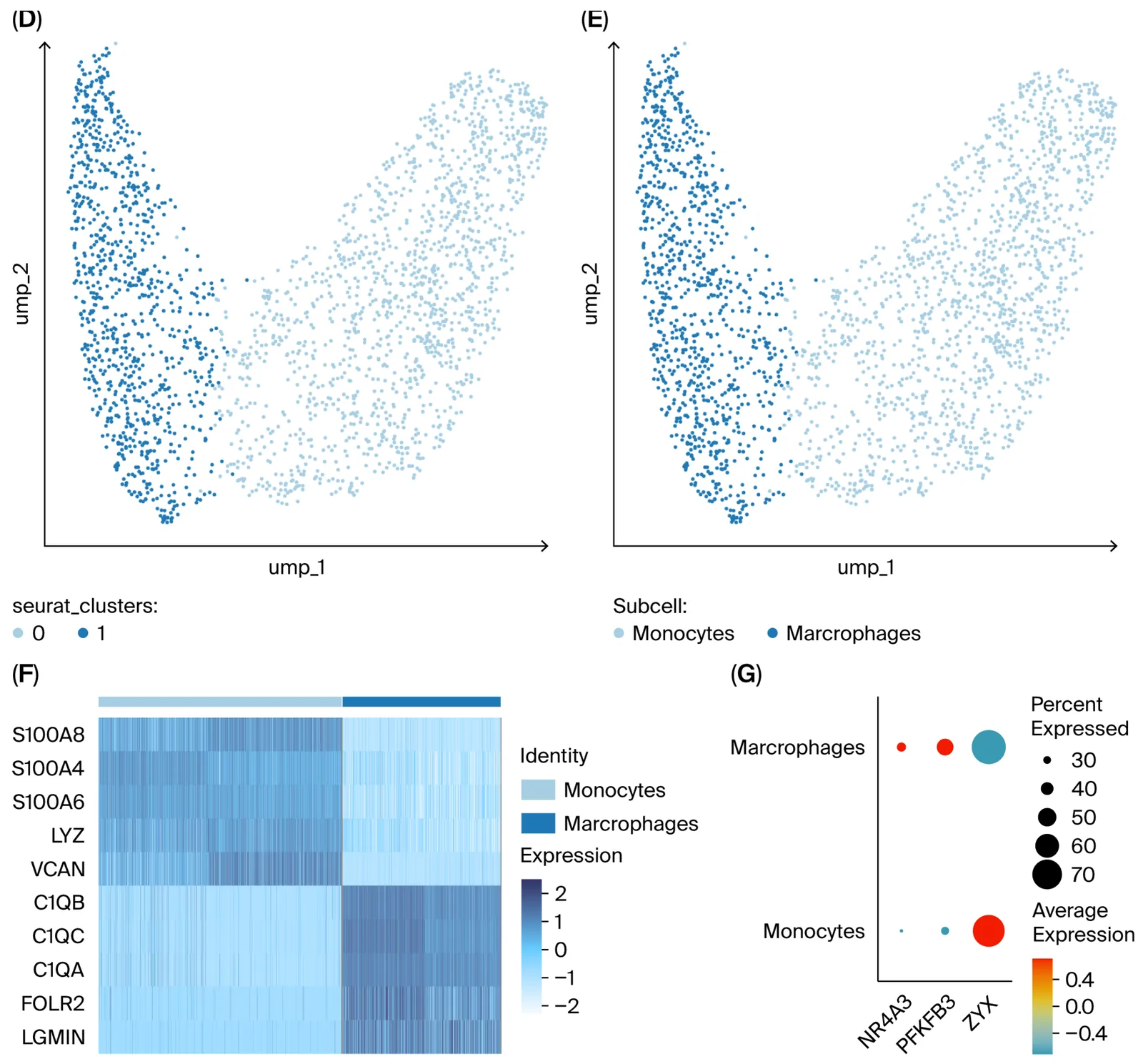
Validation of the optimal feature genes at the single-cell level. (**A**) Dot plot showing the expression of NR4A3, PFKFB3, and ZYX at the scRNA-seq level. (**B**,**C**) UMAP plot and density ridge plot showing the density of NR4A3, PFKFB3, and ZYX at the scRNA-seq level. (**D**–**F**) UMAP plots of two sub-clusters of monocytes and macrophages obtained from further clustering analysis. (**G**) Dot plot showing the expression of NR4A3, PFKFB3, and ZYX in monocytes and macrophages.

To further clarify the specific cell types in which the three optimal characteristic genes function, we conducted single-cell level validation. The results demonstrate that PFKFB3 and ZYX were highly expressed in mononuclear phagocytes, whereas NR4A3 was expressed in mononuclear phagocytes and B cells (Figure 6A,C). The UMAP visualization results were consistent with the previous analysis (Figure 6B). Furthermore, dimension reduction analysis of mononuclear phagocytes was also conducted, and the two sub-clusters of monocytes and macrophages were annotated (Figure 6D–F). In parallel, we observed that NR4A3 and PFKFB3 were mainly expressed in macrophages, whereas ZYX was expressed in monocytes (Figure 6G).

### 2.6. Results of Trajectory Analysis and Cell Interactions

**Figure 7:**
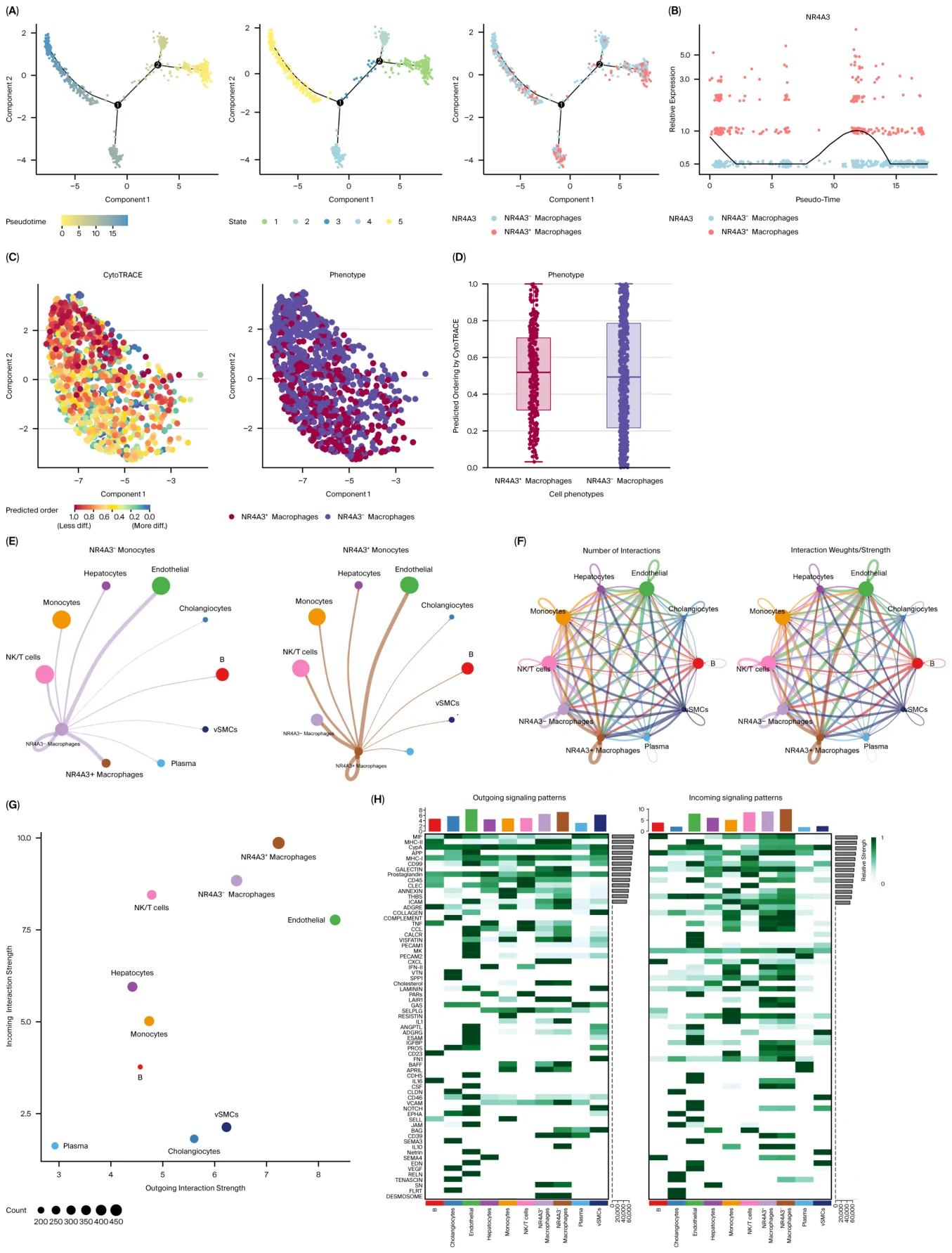

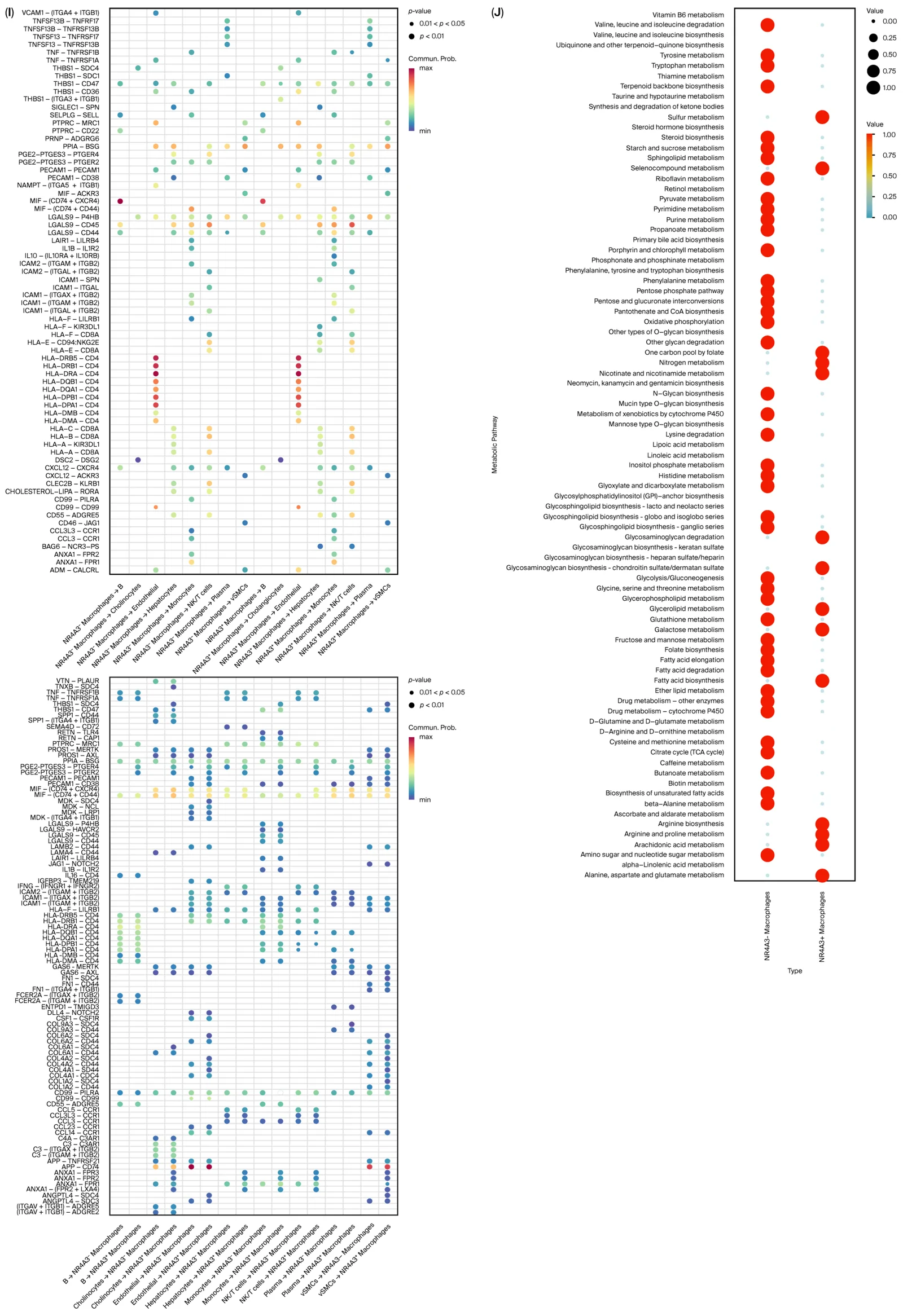
Cell trajectory and intercellular communication landscape of NR4A3+ macrophages. (**A**) Pseudo-time trajectory showing proportional changes in NR4A3+ macrophages. (**B**) Pseudo-time expression dynamics of NR4A3 along the developmental trajectory. (**C**) CytoTRACE analysis revealing the differentiation landscape of NR4A3+ macrophages. (**D**) CytoTRACE score distribution comparing NR4A3+ and NR4A3^−^ macrophages. (**E**–**G**) Cellular communication networks illustrating the quantity and intensity of interactions between NR4A3+ macrophages and other cell types. (**H**) Heatmap summarizing outgoing and incoming signaling contributions across different cell populations. (**I**) Bubble plot showing ligand–receptor interactions mediated by NR4A3+ macrophages. (**J**) Metabolic pathway enrichment analysis of NR4A3 in macrophages.

In light of the transcriptional heterogeneity of cells in HIRI, pseudo-time analysis was employed to investigate macrophages marked by NR4A3 and PFKFB3, as well as monocytes marked by ZYX. Notably, the proportion of NR4A3+ and PFKFB3+ macrophages, along with ZYX+ monocytes, remained consistently elevated over time (Figure 7A; Supplementary Figures S1A and S2A). Figure 7B shows the relative expression of NR4A3 in the pseudo-time analysis, while the results for PFKFB3 and ZYX are presented in Supplementary Figures S1B and S2B, respectively. These findings provide insights into the transcriptional heterogeneity of monocytes and macrophages in HIRI. CytoTrace analysis revealed that NR4A3+/PFKFB3+ macrophages and ZYX+ monocytes exhibited higher scores compared to their NR4A3^−^, PFKFB3^−^, and ZYX^−^ counterparts (Figure 7C,D; Supplementary Figures S1C,D and S2C,D). Since higher CytoTRACE scores indicate a less differentiated cellular state, these findings suggest that NR4A3+/PFKFB3+ macrophages and ZYX+ monocytes tend to retain a relatively immature, less-differentiated phenotype compared with their negative counterparts.

To further explore differences in intercellular communication between NR4A3^+^ and NR4A3^−^ macrophages, we examined receptor and ligand expression profiles. The results indicate that NR4A3^+^macrophages exhibited higher signal transduction efficiency than NR4A3^−^macrophages, and the same result was obtained for PFKFB3+ macrophages and ZYX+ monocytes (Figure 7E–G; Supplementary Figures S1E–G and S2E–G). Figure 7H reveals that NR4A3+ macrophages exhibited enhanced intercellular communication, with distinct outgoing (such as GALECTIN, ICAM, and VISFATIN) as well as incoming signaling patterns (such as COLLAGEN, SPP1, and FN1). Figure 7I reveals that NR4A3+ macrophages mediated intercellular interactions with diverse cell types through multiple receptor–ligand pairs, including LGALS9−CD45, HLA−DRB5−CD4, and APP−CD74. Similar findings were observed in PFKFB3+ macrophages (Supplementary Figure S1H,I). Meanwhile, ZYX+ monocytes showed distinct outgoing (ADGRE, APRIL, and VCAM) and incoming signaling patterns (GALECTIN, SPP1, FN1, and CD23) (Supplementary Figure S2H). Additionally, these cells engaged in key ligand–receptor interactions, including MIF–(CD74 + CXCR4) and APP–CD74 (Supplementary Figure S2I). The results of metabolic pathway analysis indicate that NR4A3^+^/PFKFB3^+^ macrophages and ZYX^+^ monocytes exhibit different heterogeneity characteristics compared to NR4A3^−^/PFKFB3^−^ macrophages and ZYX^−^ monocytes (Figure 7J; Supplementary Figures S1J and S2J).

### 2.7. Transcriptomic Alterations Following Virtual NR4A3 Knockout

**Figure 8:**
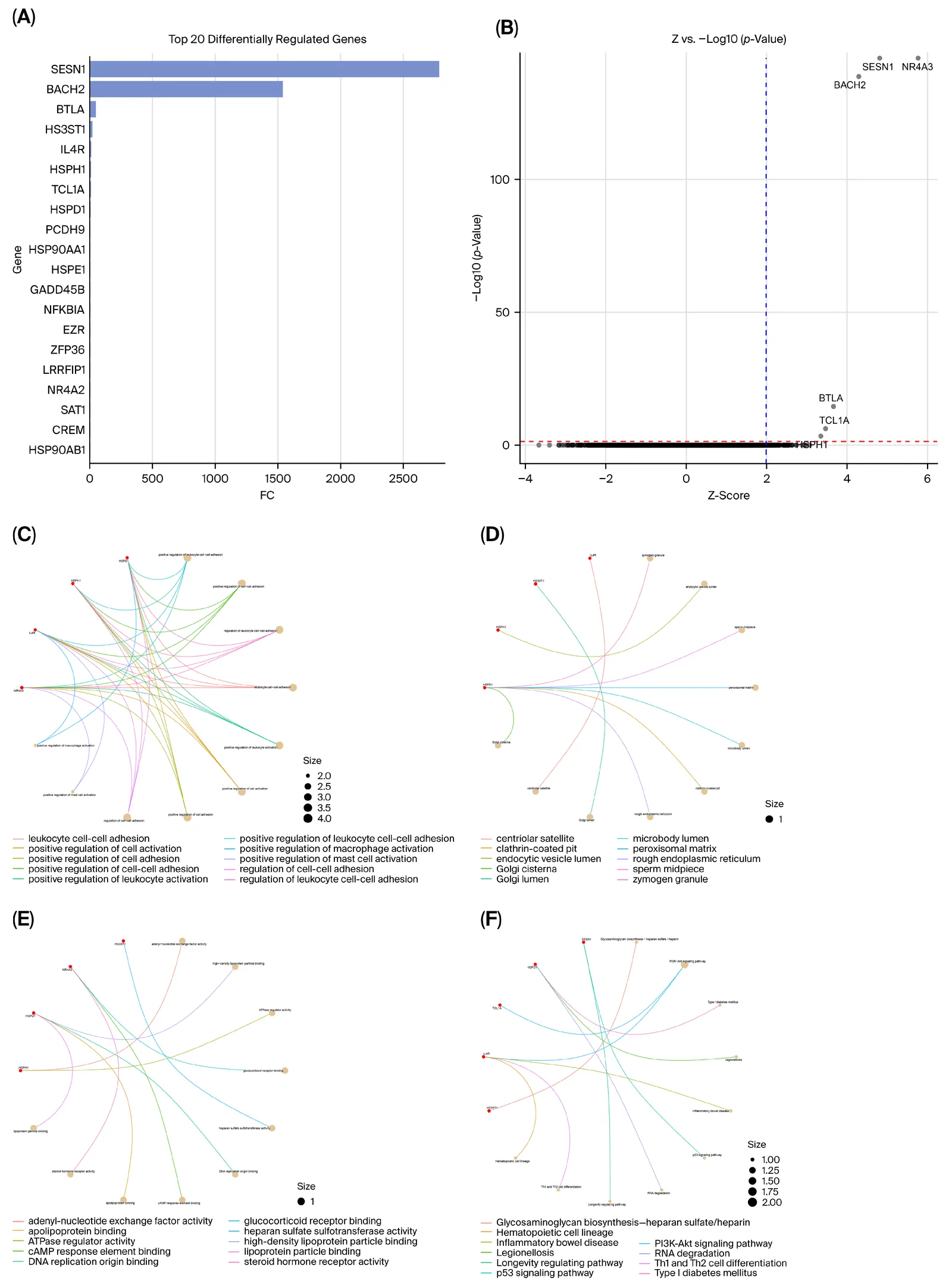
Virtual knockout perturbations and functional enrichment of NR4A3. (**A**) The top 20 differentially expressed genes with the most significant changes after NR4A3 virtual knockout (sorted by the magnitude of expression change). (**B**) Scatter plot based on Z-score and multiple correction significance analysis. (**C**–**E**) Gene Ontology (GO) enrichment pathway of the differentially expressed genes. BP, biological process; CC, cellular component; MF, molecular function. (**F**) Kyoto Encyclopedia of Genes and Genomes enrichment pathway of the differentially expressed genes.

Given that the SHAP values identified NR4A3 as the most influential predictor, we conducted an in vitro knockout analysis using “scTenifoldKnk” to assess the overall impact of NR4A3 on the transcriptional regulatory network. To identify potential downstream targets affected by NR4A3 deficiency, we performed a ranking analysis of differentially expressed genes. The NR4A3 depletion led to extensive transcriptional changes, with the top significantly regulated genes including SESN1, BACH2, BTLA, and TCL1A (Figure 8A,B). Subsequently, we performed GO and KEGG enrichment analyses on the top differentially expressed genes to elucidate their functional roles in biological processes. In terms of the biological process (BP) category, these genes exhibited significant upregulation in leukocyte cell–cell adhesion and positive regulation of cell activation (Figure 8C). Within the cellular component (CC) category, there was a marked correlation with centriolar satellites and zymogen granules (Figure 8D). Regarding molecular function (MF), these genes were closely associated with adenyl-nucleotide exchange factor activity and ATPase regulator activity (Figure 8E). KEGG enrichment analyses indicated that these genes were mainly enriched in the PI3K−Akt signaling pathway and glycosaminoglycan biosynthesis–heparan sulfate/heparin (Figure 8F).

### 2.8. NR4A3-Targeting Inhibitors and Molecular Dynamic Validation

**Figure 9:**
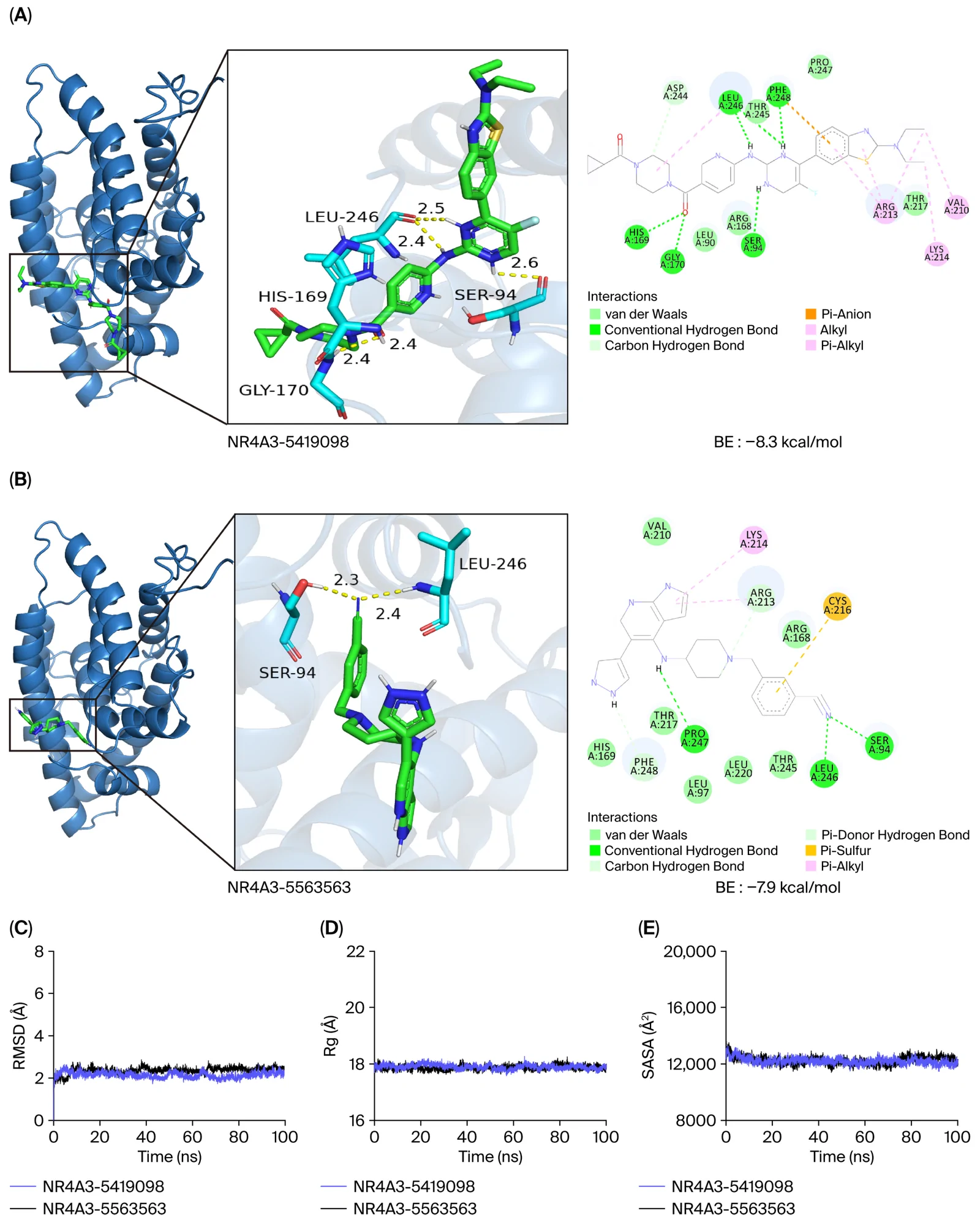

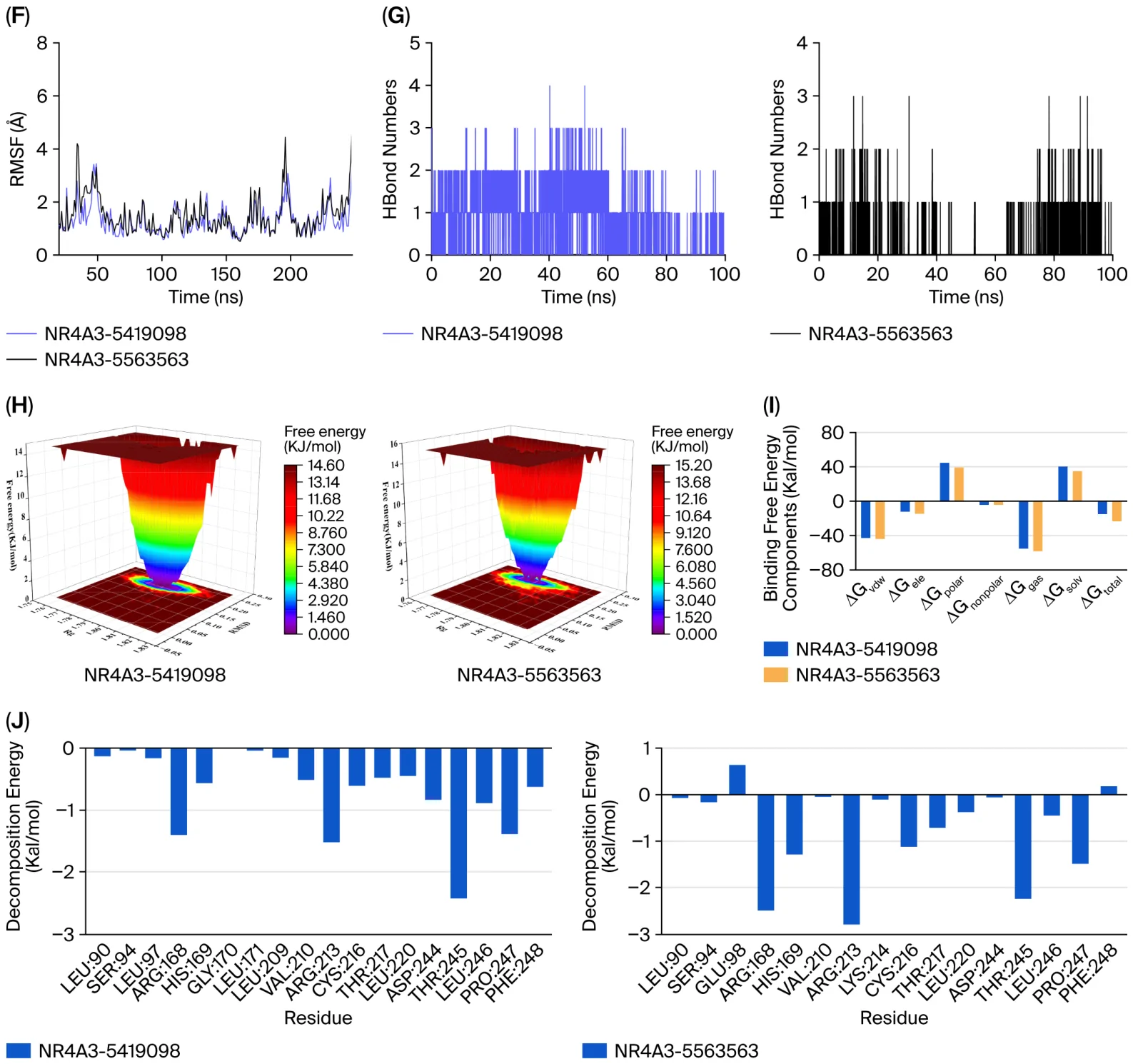
Molecular docking and molecular dynamic simulation results of NR4A3 with the predicted drugs. (**A**) Docking result of the NR4A3 and CHEMBL5419098 complex. (**B**) Docking result of the NR4A3 and CHEMBL5563563 complex. (**C**) RMSD values of the two complexes. (**D**) Radius of gyration (Rg) values of the two complexes. (**E**) Solvent-accessible surface area (SASA) values of the two complexes. (**F**) Root-mean-square fluctuation (RMSF) values of the two complexes. (**G**) Number of hydrogen bonds in the two complexes. (**H**) Free energy landscapes of two complexes. (**I**) Binding free energies of the two complexes. (**J**) Decomposition energy of the two complexes. Detailed procedures for molecular docking and molecular dynamic simulations are described in the Materials and Methods Section.

To identify potential NR4A3 targets, we analyzed key inhibitors using the ChEMBL online tool. Two compounds with quantitative pIC50 were identified: CHEMBL5419098 (IC50 0.69 nM) and CHEMBL5563563 (IC50 3.8 μM). An independent IC50 specificity search confirmed that CHEMBL5419098 was the most potent among all quantitative records. Both compounds were advanced to molecular docking, CHEMBL5419098 as a high-potency reference and CHEMBL5563563 as a structurally distinct comparator. The docking analysis showed that both compounds could be accommodated within the predicted NR4A3 binding pocket, forming hydrogen bonds and hydrophobic interactions, with docking scores of −8.3 kcal/mol for CHEMBL5419098 and −7.9 kcal/mol for CHEMBL5563563 (Figure 9A,B). To assess the dynamic stability of these interactions, we performed molecular dynamic simulations on the NR4A3-CHEMBL5419098 and NR4A3-CHEMBL5563563 complexes. After an initial equilibration phase, both systems reached relatively stable conformational states, as reflected by the RMSD and radius of gyration (Rg) trajectories (Figure 9C,D). The solvent-accessible surface area (SASA) initially decreased and then fluctuated within a narrower range (Figure 9E). RMSF analysis indicated that larger fluctuations were mainly confined to flexible regions, whereas the putative binding regions remained relatively stable (Figure 9F). Hydrogen bond tracking revealed persistent hydrogen bonds within both complexes throughout most of the simulation (Figure 9G). The free energy landscape was dominated by a low-energy basin, consistent with a favorable conformational state (Figure 9H). MM/PBSA analysis further revealed favorable overall binding, with nonpolar and van der Waals interactions contributing predominantly to complex stabilization. Per-residue decomposition further pinpointed residues that notably stabilized CHEMBL5419098 and CHEMBL5563563 within the binding pocket (Figure 9I,J). Collectively, these in silico results indicated that CHEMBL5419098 and CHEMBL5563563 possessed favorable binding affinity for NR4A3 and stable simulation dynamics. Nevertheless, additional experimental validation was needed to verify their direct interaction with NR4A3.

### 2.9. Validation of Global Lactylation Level and Key LRGs in a Mouse HIRI Model

**Figure 10:**
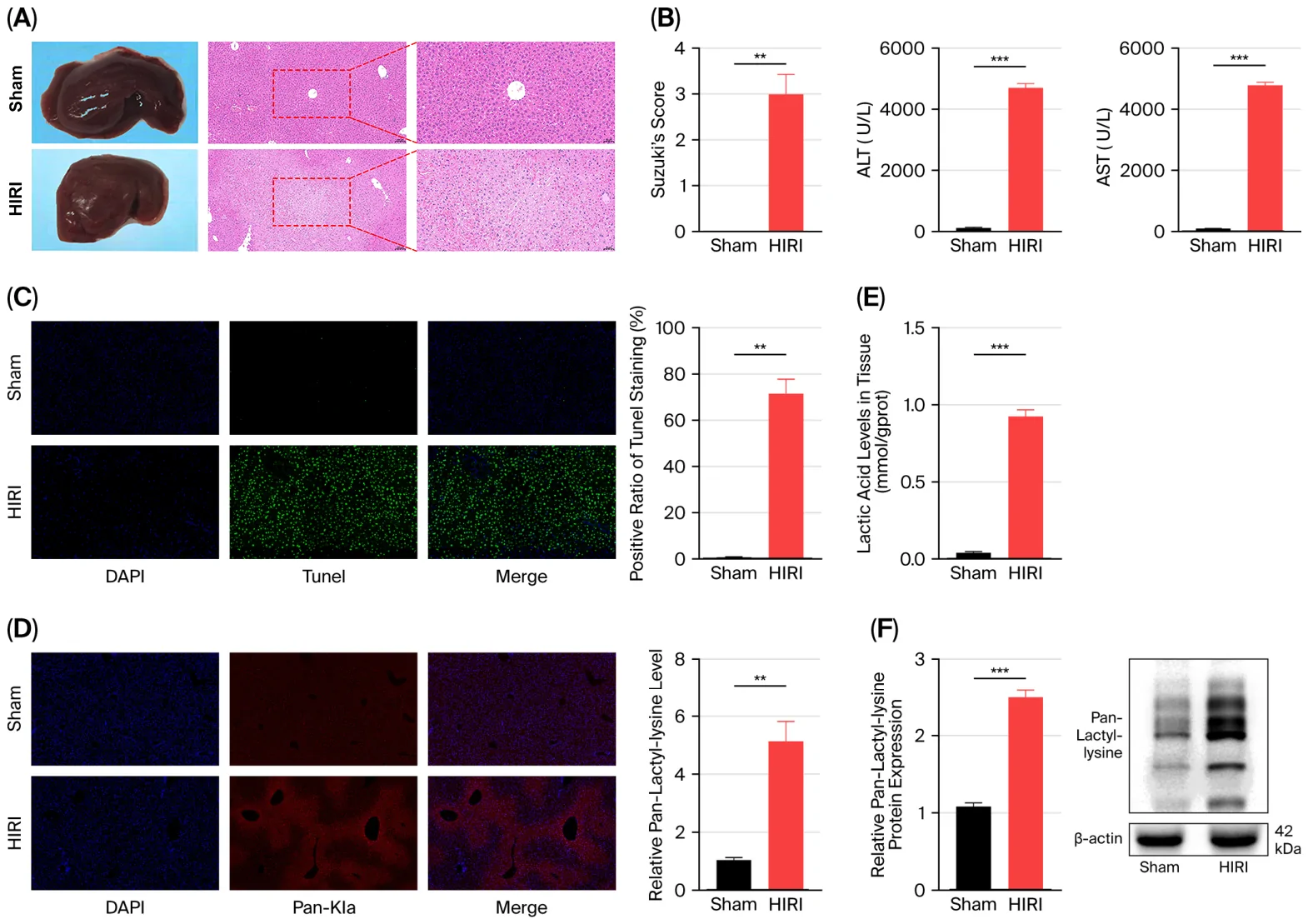

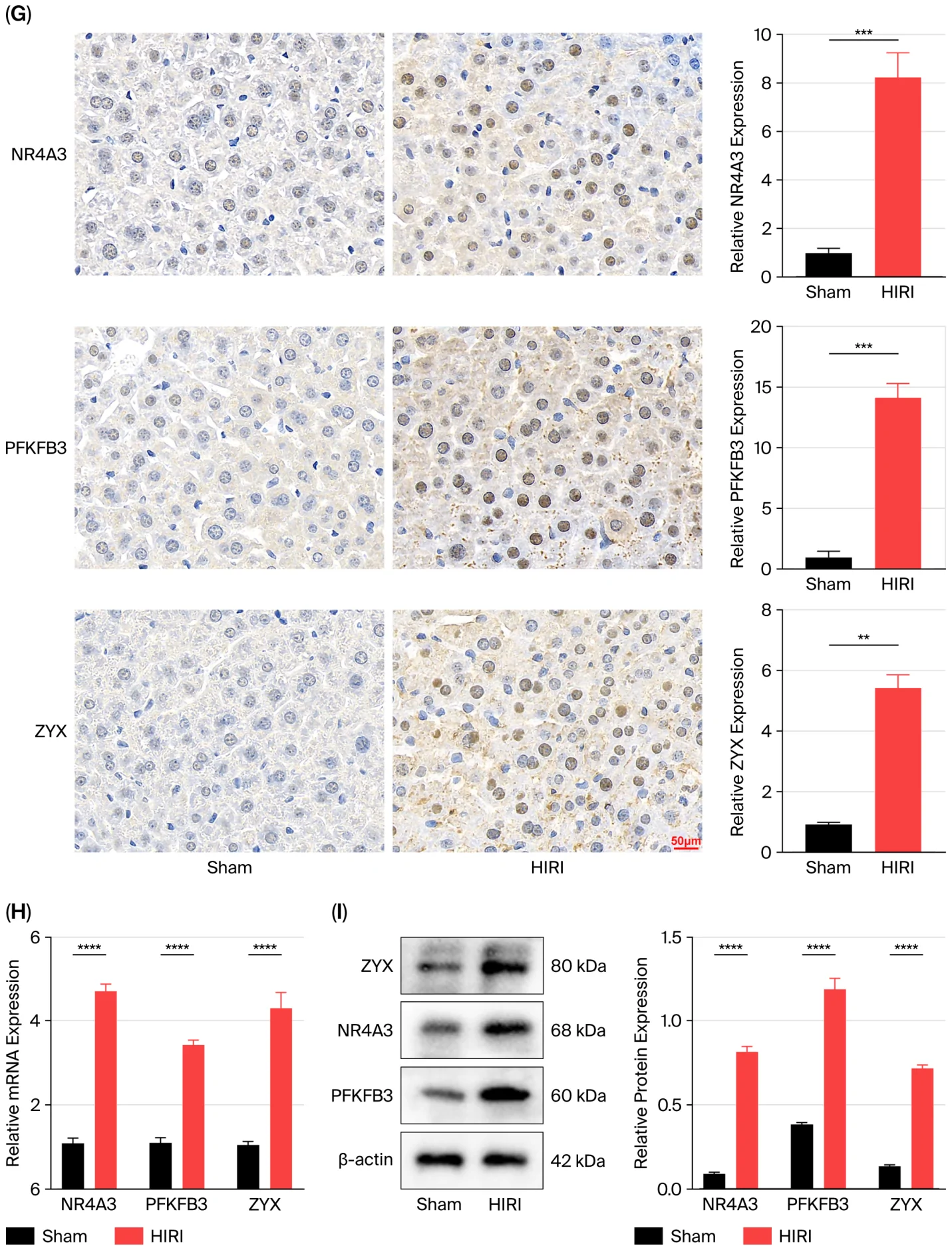
Experimental validation of global protein lactylation and key LRGs in the mouse HIRI mode. (**A**) General images of mice and hematoxylin and eosin in the sham and HIRI groups. (**B**) Liver damage, evaluated by Suzuki’s score (*p* = 0.007). Serum ALT (*p* = 0.0002) and AST (*p* = 0.0002) levels in the sham and HIRI groups. n = 5 mice per group. Data are presented as mean ± SD; ** *p*  <  0.01 and *** *p*  <  0.001 by Student’s two-tailed unpaired *t*-test. (**C**) TUNEL staining of liver specimens in the sham and HIRI groups. ** *p* <  0.01. Scale bar = 100 μm. (**D**) The protein lactylation levels in liver tissues of sham and HIRI group mice were evaluated by immunofluorescence analysis. Scale bar = 100 μm. ** *p*  <  0.01. (**E**) Lactic acid levels in tissues between the sham and HIRI groups. *** *p* <  0.001. (**F**) Western blot analysis and quantitative analysis of Pan-Lactyl-lysine protein expressions in liver tissues. *** *p*  <  0.001. (**G**) The protein lactylation levels in liver tissues of sham and HIRI group mice were assessed by immunohistochemical analysis. Scale bar = 50 μm. ** *p*  <  0.01; *** *p*  <  0.001. (**H**) Comparison of the three LRG mRNA expression levels in liver tissues between the sham and HIRI groups. n = 5 mice per group. Data are presented as mean ± SD; **** *p* < 0.0001 by Student’s two-tailed unpaired *t*-test. (**I**) Western blot analysis and quantitative analysis of the three LRG protein expressions in liver tissues between the sham and HIRI group. n = 5 mice per group. Data are presented as mean ± SD; **** *p*< 0.0001 by Student’s two-tailed unpaired *t*-test.

To investigate the pathological impact of lactate metabolic reprogramming and lactylation on HIRI, we established a murine HIRI model. Histopathological examination and TUNEL staining exhibited severe sinusoidal congestion, edema, vacuolization, and apoptosis, along with markedly elevated serum ALT and AST levels (Figure 10A–C). We next assessed lactate metabolism and the global lactylation profile in this model. Western blotting and immunofluorescence revealed increased protein lactylation in HIRI tissues, accompanied by pronounced lactate accumulation (Figure 10D–F), collectively indicating enhanced lactylation during HIRI. We then evaluated the expression of the three key LRGs in the mouse model. RT-qPCR, immunohistochemistry, and Western blotting consistently showed that NR4A3, PFKFB3, and ZYX were upregulated at both the mRNA and protein levels in HIRI tissues (Figure 10G–I). Collectively, these results indicate that the key LRGs identified in our mouse models are potential therapeutic targets for mitigating HIRI.

## 3. Discussion

Liver transplantation (LT) remains the only effective therapeutic option for end-stage liver disease and liver cancer. However, HIRI represents a serious complication and a major risk factor for early graft failure and hepatic dysfunction after LT while also contributing substantially to donor organ shortage [3]. Mitigating HIRI could broaden the use of marginal allografts and improve post-transplant outcomes. Lactylation refers to the transfer of lactyl groups to lysine residues on proteins, modulating their activity and function. Histone lactylation serves as a key epigenetic regulator of pro-inflammatory gene expression in macrophages and cancer cells, whereas the lactylation of non-histone proteins modulates enzymatic activity to promote metabolic stress adaptation [19]. Accumulating evidence indicates that lactate metabolism is critically involved in regulating cellular responses to HIRI stress. Nevertheless, the underlying regulatory mechanisms and the key genes involved remain poorly understood. In this study, we systematically analyzed lactylation activity across multiple cell types in HIRI tissues by integrating multi-omics data with machine learning algorithms, providing novel insights into the metabolic contributions to the pathogenesis of HIRI.

In this study, we applied single-cell sequencing and multiple scoring algorithms to evaluate LRG activity across distinct cell populations in HIRI. Through the integration of differential expression and WGCNA analyses, we identified three characteristic genes that were markedly upregulated and closely associated with dysregulated lactylation. Single-cell RNA-seq data further confirmed that NR4A3 and PFKFB3 were elevated in macrophages, whereas ZYX was upregulated in monocytes. Subsequently, we found that the diagnostic values of these key genes showed high predictive accuracy at the bulk-RNA level, and NR4A3 made the greatest contribution to the model according to SHAP analysis. A series of molecular biology experiments further validated these bioinformatic findings. We also conducted an in vitro knockout analysis using the scTenifoldKnk tool to assess the overall impact of NR4A3 on the transcriptional regulatory network. Additionally, to evaluate the therapeutic potential of targeting NR4A3, we analyzed key inhibitors using the ChEMBL online tool, which indicated that CHEMBL5419098 and CHEMBL5563563 had favorable binding affinity for NR4A3 and exhibited stable dynamic profiles during simulation. In conclusion, this study offers novel perspectives on the molecular mechanisms driving HIRI and uncovers potential targets for therapeutic intervention.

The nuclear receptor subfamily 4, group A, member 3 (NR4A3), an orphan nuclear receptor, plays a crucial role in mediating immune cell homeostasis, the inflammatory response, tumor cell proliferation, and cellular metabolism as a molecular switch or transcription factor [20]. Notably, emerging evidence indicates that NR4A3 is rapidly activated and influences the inflammatory process in various pathological conditions, acting as an early stress factor. Alonso et al. demonstrated that the overexpression of NR4A3 promotes reactive oxygen species (ROS) production in human vascular smooth muscle cells (VSMCs), leading to the disruption of redox homeostasis [21]. In acute pancreatitis, NR4A3 expression is markedly elevated, and its knockdown alleviates pancreatitis-induced injury via the regulation of Btg2, thereby reducing oxidative stress, mitochondrial damage, and apoptosis [22]. Additionally, NR4A3 can inhibit the proliferation of lung cancer cells by directly binding to p53 and upregulating pro-apoptotic genes, thereby promoting cell apoptosis [23]. In cellular metabolism, Ma et al. identified NR4A3-mediated histone lactylation as a novel metabolome–epigenome signaling cascade that contributes to the pathogenesis of medial arterial calcification [24]. Moreover, Liu et al. showed that the cuproptosis induced by myocardial ischemia–reperfusion in aged hearts was mainly concentrated in NR4A3-positive cardiomyocytes and RGCC-positive capillary endothelial cells [25]. To the best of our knowledge, the association between NR4A3 and HIRI has not been previously documented, and its involvement in lactate metabolism remains poorly understood. In our study, we observed that NR4A3 was highly expressed in macrophages, prompting us to further explore its regulatory mechanism.

6-phosphofructo-2-kinase/fructose-2,6-biphosphatase 3 (PFKFB3), a bifunctional enzyme regulating glycolysis, serves as a key determinant of glycolytic rates by modulating fructose-2,6-bisphosphate (F-2,6-BP) levels, thereby allosterically activating the rate-limiting enzyme 6-phosphofructokinase-1 (PFK-1) [26]. Increasing evidence indicates that PFKFB3 is ubiquitously expressed and critically involved in inflammatory response, immune metabolism, and angiogenesis. Zhang et al. demonstrated that PFKFB3 is upregulated during acute pancreatitis progression and aggravates the severity of alcohol-associated acute pancreatitis via IP_3_R-mediated Ca^2+^ elevation [27]. In acute myocardial infarction, the suppression of PFKFB3 in macrophages enhances vascular endothelial cell growth factor C (VEGF-C) production, thereby facilitating lymphangiogenesis and attenuating the inflammatory response to ischemia–reperfusion (I/R) injury [28]. Additionally, increased PFKFB3 is closely associated with the excessive inflammatory response and high mortality in sepsis [29]. Lactate, a byproduct of glycolysis, has been proven to modulate gene expression through histone lactylation. PFKFB3 expression correlated positively with urinary lactate and renal fibrosis severity in chronic kidney disease patients. Mechanistically, tubular PFKFB3 deletion attenuated renal inflammation and fibrosis through promoting histone lactylation-mediated NF-kB family activation [30]. Our study indicates that PFKFB3 was highly expressed and localized to macrophages, suggesting that its inhibition may serve as a therapeutic strategy to reduce inflammation and alleviate HIRI.

Zyxin (ZYX) is a zinc-binding phosphoprotein that concentrates at focal adhesions and along the actin cytoskeleton, playing a critical role in cell proliferation, signal transduction, and apoptosis, with its dysregulation closely linked to multiple diseases [31]. In acute pancreatitis, the downregulation of ZYX suppresses TGF-β/SMAD signaling, thereby inhibiting acinar cell ferroptosis and ameliorating pancreatic injury [32]. In skin fibrosis, Zyxin inhibition effectively attenuates fibrosis by regulating the FAK/PI3K/AKT and TGF-β signaling pathways via integrins [33]. In prostate carcinoma, Zyxin directly binds to chromosomal DNA and is associated with mitochondrial integrity and apoptosis [34]. Additionally, a previous bioinformatic analysis indicated that Zyxin was closely linked to lactylation and macrophage polarization, implicating these biological processes as potentially critical mediators in inflammatory bowel disease [35]. In our study, we found that ZYX was highly expressed and predominantly localized in monocytes. Targeting ZYX in these cells to modulate lactylation may serve as a promising therapeutic approach for HIRI, yet the potential mechanism warrants further exploration.

Furthermore, Cellchat analysis revealed that NR4A3+ macrophages and ZYX+ monocytes exhibited distinct intercellular communication patterns in the HIRI microenvironment. In our study, we found that NR4A3+ macrophages mediated intercellular interactions with diverse cell types through multiple receptor–ligand pairs, including LGALS9-CD45, HLA-DRB5-CD4, and APP-CD74. Similarly, ZYX^+^ monocytes displayed prominent cell–cell crosstalk mediated by key inflammatory ligand–receptor pairs, including APP-CD74. The LGALS9-CD45 axis has previously been reported in single-cell analyses of cirrhotic liver, where macrophage-derived LGALS9 modulates T-cell function via surface CD45 to shape intrahepatic immune responses [36]. Moreover, the APP-CD74 axis has been shown to mediate endothelial cell–macrophage communication and promote kidney injury and fibrosis [37], suggesting that this pathway may also contribute to organ injury in HIRI. Given the important role of lactate metabolism in immune regulation, the distinct communication patterns of NR4A3^+^ macrophages and ZYX^+^ monocytes may partly reflect lactylation-mediated modulation of receptor and ligand expression. These findings suggest that NR4A3^+^ macrophages and ZYX^+^ monocytes may actively coordinate with other cell populations through these ligand–receptor pairs to amplify the inflammatory cascade during HIRI. Future studies are warranted to experimentally validate these ligand–receptor interactions and their downstream signaling pathways in vivo.

Despite the novel insights provided by this study, several limitations should be acknowledged. First, one major limitation of this study is that the scRNA-seq dataset (GSE171539) applied for cell-type-specific analysis contained no biological replicates (n = 1 per group). Although our filtered dataset was of high quality (7963 cells), the lack of replicates may reduce the generalizability of our cell-type-specific results. Larger scRNA-seq cohorts with biological replicates are therefore required in future studies to validate the cell-type heterogeneity of lactylation-related genes in HIRI. Second, the initial lactylation-related gene list was retrieved from previous cancer-focused publications (hepatocellular carcinoma and gastric cancer). This published LRG list was used as a pre-defined candidate gene set for exploratory bioinform screening, rather than a gold-standard gene panel specific for HIRI. Although multi-step bioinformatic filtering and experimental validation were applied to refine candidate genes, potential inherent bias from the original gene set cannot be completely excluded. Future investigations should establish a lactylation-related gene panel directly based on HIRI samples to further improve the reliability of signature screening. Third, although our results indicate that CHEMBL5419098 and CHEMBL5563563 bind favorably to NR4A3 with stable dynamics, these findings serve only as prioritization evidence and require further validation through biophysical and cell-based assays to confirm NR4A3 as a bona fide target in HIRI. Fourth, we acknowledge that our animal experiments only compared the sham and HIRI groups, demonstrating an association rather than a causal role for these three candidate genes. The molecular mechanisms underlying their contribution to HIRI progression remain elusive. Further investigation is warranted to delineate the specific signaling pathways and cellular processes regulated by these genes.

## 4. Materials and Methods

### 4.1. Data Acquisition

In this study, scRNA-seq data for HIRI was obtained from the GSE171539 database, which includes 1 preprocurement sample and 1 post-reperfusion sample. Two bulk RNA-seq datasets (GSE12720 and GSE112713) were collected from the GEO database. The training dataset was derived from GSE12720 and comprised pre-transplant (n = 21) and post-transplant (n = 21) samples. The GSE112713 dataset consisted of pre-transplant (n = 11) and post-transplant (n = 11) samples for validation. The lactylation-related genes (LRGs) were derived from the previous literature, and a total of 371 LRGs were identified [38,39] (Table S1).

### 4.2. ScRNA-Seq Data Processing

Raw scRNA-seq data were processed using the Seurat R package (Seurat 5.2.1) [40] to generate Seurat objects. Cells meeting the following quality criteria were retained for analysis: detected genes between 200 and 7000, mitochondrial gene content below 20%, and red blood cell gene expression below 3%. After filtration, 7963 high-quality cells were obtained. We selected 2000 highly variable genes via the FindVariableFeatures function and scaled the data using ScaleData. Dimensionality reduction was performed with principal component analysis (PCA), retaining the top 30 principal components. Batch effects between samples were corrected using Harmony (v0.1.1) [41]. Finally, we conducted cell clustering with FindNeighbors and FindClusters and visualized the results using two-dimensional UMAP projection [42].

### 4.3. Signaling Pathway Score

In this study, we leveraged a comprehensive approach combining five established algorithms—AUCell [24], UCell [25], Singscore [26], ssGSEA [27], and AddModuleScore [28]—to assess lactylation activity at the single-cell level and calculate the total lactylation activity. Cells were categorized into high- and low-lactylation groups based on the median score. Subsequently, the “FindMarkers” function was used to identify differentially expressed genes (DEGs) involved in the upregulation of lactylation activity [43].

### 4.4. High-Dimensional Weighted Correlation Network Analysis

We performed weighted gene co-expression network analysis (hdWGCNA) using the “hdWGCNA” package (version 0.4.12) [44]. The “pickSoftThreshold” function was used to determine the optimal soft threshold for constructing a weighted gene co-expression network. Based on this threshold, the correlations between genes were calculated to construct a co-expression matrix. Genes were then hierarchically clustered into distinct co-expression modules using the “hclust” function. The correlation between the modules and cell clusters was subsequently analyzed using module Eigengenes to determine the most relevant modules.

### 4.5. Enrichment Analysis

Functional enrichment analyses, including Gene Ontology (GO) and KEGG pathway analyses, were performed on the candidate genes via clusterProfiler [45]. The GO terms covered three domains: BP, CC, and MF.

### 4.6. Simulated Knockout Analyses of Key Genes

To further investigate the potential impact of key genes on the pathogenesis of HIRI, we conducted single-cell gene regulatory networks using the scTenifoldKnk R package based on scRNA-seq data [46]. Then, we performed in silico gene knockout experiments to pinpoint genes that were significantly perturbed by the virtual knockout (*p* < 0.05). Additionally, Gene Ontology (GO) and KEGG pathway enrichment analyses were carried out for these perturbed genes to elucidate the molecular mechanisms of the core targets in HIRI.

### 4.7. Machine Learning Modeling and SHAP Interpretability Analysis Identify Core Genes

To construct the best model, five machine learning algorithms were adopted for feature selection and hub gene identification, including least absolute shrinkage and selection operator (LASSO), Random Forest (RF), Generalized Boosted Regression Modeling (GBM), Boruta, and Decision Tree (DT). LASSO regression analysis tools enhance the predictive power of statistical models by simultaneously performing variable selection and regularization. Random Forest (RF) is a widely employed ensemble learning method for diverse prediction tasks, which constructs multiple decision trees using random sampling and feature selection, with predictions based on voting or averaging. The Boruta algorithm determines feature importance by comparing original features with randomly permuted counterparts. It can effectively handle high-dimensional data without requiring extensive preprocessing. Gradient Boosting Machine (GBM) builds a powerful predictive model by combining multiple weak learners, while Decision Tree (DT) partitions the data to maximize the homogeneity of the subsets. By integrating these complementary machine learning strategies—each with its distinct feature selection and validation frameworks—we systematically identified the most critical hub genes associated with lactylation activity.

Shapley Additive exPlanations (SHAP) is a game-theoretic approach used to explain the output of any machine learning model [18]. It calculates the Shapley value for each feature, which represents its precise marginal contribution to a specific prediction, thereby quantifying feature importance at the individual prediction level. To address the interpretability challenge (the “black box” nature) of complex machine learning models, we implemented the Shapley Additive exPlanations (SHAP) algorithm to deconstruct and quantify the contribution of individual features to each predictive outcome. This interpretable framework facilitated the inference of potential biological mechanisms underlying the model’s decisions.

### 4.8. Trajectory Analysis

To reconstruct cellular developmental trajectories, we divided cells into two groups according to the expression status of the feature genes of interest. The Monocle2 algorithm was then applied to infer pseudo-time trajectories using the gene–cell–matrix extracted from the Seurat subset (with scaled UMI counts) as input under default parameters [47]. For gene set enrichment analysis, we used relevant marker gene sets from the Molecular Signatures Database (MSigDB). The GSVA package was run with default settings to compute the average expression level of each gene set across cell subtypes. Differences in activity derived from this analysis were applied to quantify pathway activity variations among distinct cell subtypes. To further corroborate and enhance the results from Monocle2’s trajectory analysis, we also employed CytoTRACE (Cellular Trajectory Reconstruction Analysis) with default parameters [48]. This algorithm was based on the well-established principle that transcriptional diversity decreases during cellular differentiation. By applying this metric to our scRNA-seq data, CytoTRACE predicted the differentiation state of individual cells, thereby providing a complementary approach to confirm and extend the trajectory inference obtained through Monocle2.

### 4.9. Cell–Cell Interaction Analysis

Based on the annotated scRNA-seq dataset, we employed the CellChat R package (version 2.2.0) [49] to systematically analyze intercellular communication networks, with a focus on ligand–receptor-mediated interactions between distinct cell types. Initially, we constructed cell–cell communication networks using CellChat. To visualize intercellular communication patterns, the netVisual_circle function was employed to display the number and strength of interactions between the target cell cluster and other clusters. Subsequently, the netVisual_bubble function was used to generate bubble plots, highlighting significant ligand–receptor interactions involving the target cluster and underscoring key communication pathways across different cell types.

### 4.10. Animals and Treatment

Eight-week-old male C57BL/6J mice, weighing 20–25 g, were obtained from Jiangsu GemPharmatech Company. The mice were maintained in a specific pathogen-free (SPF) environment and housed in accordance with laboratory animal care principles (22 ± 2 °C, 12 h light/dark cycle, and 50 ± 10% humidity). The animals were monitored daily for signs of pain or distress. Following a one-week adaptation period, the mice were randomly assigned to two groups (N = 5 in each group): sham and HIRI. Sample size (n = 5 per group) was determined based on previous studies and pilot experiments in our laboratory. No a priori power calculation was performed. Randomization was performed using a computer-generated random number sequence. Model: Mice were anesthetized via intraperitoneal injection of pentobarbital sodium (50 mg/kg). A model of 70% partial hepatic ischemia was then established by occluding the portal vein and hepatic artery to the left and median liver lobes, as previously described [50]. The hepatic pedicle was clamped for 60 min, followed by clamp removal to allow for reperfusion. After 6 h of reperfusion, blood serum and liver tissue samples were collected for subsequent analysis. The sham mice underwent the same surgical procedure, including isolation of the portal vein and hepatic artery without vascular occlusion. Humane endpoints were defined as follows: (1) severe lethargy or unresponsiveness to gentle stimulation, (2) labored breathing, (3) inability to ambulate, or (4) weight loss exceeding 20% of baseline body weight. Animals meeting any of these criteria would have been humanely euthanized with an overdose of pentobarbital sodium. No animals reached the humane endpoints during this study. All surgical procedures were performed by the same surgeon. No specific inclusion or exclusion criteria were applied. All mice that successfully underwent the surgical procedure were included in the analysis. No animals were excluded from the analysis. The surgeon was not blinded to group allocation due to the nature of the surgical procedure. However, all outcome assessments, including histopathological examination, Western blot analysis, and RT-qPCR, were performed by investigators who were blinded to the experimental groups.

### 4.11. Liver Injury Analysis, Quantitative Real-Time PCR, and Western Blot

To assess the extent of hepatic injury, serum levels of alanine aminotransferase (ALT) and aspartate aminotransferase (AST) were measured using standard biochemical assays. Additionally, hematoxylin and eosin (H&E) staining was performed on liver tissue sections for histopathological evaluation. Total RNA of liver samples was extracted using the RNA Extraction Reagent (Vazyme, Nanjing, China) according to the manufacturer’s protocol, and the relative mRNA expression of featured key genes was further evaluated.

Protein extraction from tissue samples was extracted using RIPA buffer and quantified with the BCA Protein Assay Kit (Beyotime, Beijing, China). Equal amounts of protein were separated by 10% SDS–polyacrylamide gel electrophoresis (SDS-PAGE) under denaturing conditions and then electrophoretically transferred to polyvinylidene difluoride (PVDF) membranes. After blocking, membranes were incubated with specific primary antibodies and HRP-conjugated secondary antibody. Protein bands were visualized using an enhanced chemiluminescence (ECL) detection system.

### 4.12. Drug Prediction and Molecular Docking

To identify potential NR4A3 inhibitors, we performed a systematic search of the ChEMBL database using a pragmatic two-step strategy. Given the limited availability of compounds with directly reported IC50 values for NR4A3, we first applied an initial filter to retrieve records with Standard Type designated as “Inhibition,” Standard Units as “%,” and Standard Relation as “=”, excluding records with missing standard values. Compounds with measurable inhibition activity were retained as candidates for further evaluation. We then performed a confirmatory quantitative assessment for these candidates by cross-referencing their available IC50 data in the ChEMBL database, ensuring that the final selected compounds were evaluated using the gold-standard metric for inhibitor potency. For compounds where IC50 was reported as pIC50, we converted these to IC50 using the formula: IC50 = 10^−pIC50^ [51]. Two compounds were selected for docking: CHEMBL5419098 as the most potent inhibitor and CHEMBL5563563 as a structurally distinct comparator.

Molecular docking simulations were conducted to validate the predicted interactions [52]. The 3D structures of the ligand molecules were obtained from the PubChem database and subsequently prepared using Open Babel. The 3D structure of the target protein was sourced from the RCSB Protein Data Bank (PDB). Subsequent protein preparation in PyMOL (version 3.1.0) involved the removal of water molecules and non-essential co-crystallized ligands, as well as the addition of hydrogen atoms. The prepared ligands and the protein receptor were then converted to pdbqt format using AutoDockTools 1.5.7, with explicit parameterization of the protein’s active site. Docking simulations were executed with AutoDock Vina, and resulting poses with binding energies below −5.0 kcal/mol were defined as high-affinity binders. The optimal binding modes were visualized in PyMOL, and detailed interaction profiles were delineated using the Protein–Ligand Interaction Profiler (PLIP).

### 4.13. Statistical Analysis

All data are presented as the mean ± standard deviation. The normality of the data was assessed using the Shapiro–Wilk test. Since all data passed the normality test, comparisons between two groups (sham vs. HIRI) were performed using the unpaired Student’s *t*-test. The level of statistical significance was set at *p* < 0.05. All statistical analysis was performed using SPSS software (version 28.0).

## 5. Conclusions

In conclusion, this study integrates transcriptomic profiling with experimental validation to identify NR4A3, PFKFB3, and ZYX as key LRGs driving HIRI in liver transplantation. These key LRGs, identified across single-cell RNA-seq and bulk datasets as well as in murine HIRI models, are mechanistically linked to lactate-driven metabolic reprogramming and inflammatory activation. By connecting lactate metabolism to epigenetic and inflammatory networks, our findings establish a conceptual basis for therapeutic strategies targeting graft injury and transplant outcome improvement. Future work should focus on lactylation site-specific modifications and clinical evaluation of LRG-targeted interventions to enhance liver transplant outcomes.

## Data Availability

The data that support the findings of this study are available from the Gene Expression Omnibus database (https://www.ncbi.nlm.nih.gov/geo/), a publicly accessible repository maintained by the National Center for Biotechnology Information (NCBI), accessed on 4 June 2026. The specific GEO accession numbers and corresponding datasets are cited within the manuscript.

## Supplementary Materials

The following supporting information can be downloaded at: https://www.mdpi.com/article/10.3390/ijms27177965/s1.
