# Supplementary material for: Integration of Single-Cell and Bulk RNA Sequencing Data to Identify Lactylation-Related Gene Signatures in Hepatic Ischemia–Reperfusion Injury Using Machine Learning Algorithms
Source: Int J Mol Sci. 2026 Sep 7;27(17):7965. doi: 10.3390/ijms27177965 (PMC13566055; doi:10.3390/ijms27177965)
Supplement: Supplementary file 1 [file ijms-27-07965-s001.zip › Author Checklist - Full.pdf]

NOTE: Please save this file locally before filling in the table, DO NOT work on the file within your internet browser as changes will not be saved. Adobe Acrobat Reader (available free here) is recommended for completion.

The ARRIVE guidelines 2.0: author checklist

The ARRIVE Essential 10

These items are the basic minimum to include in a manuscript. Without this information, readers and reviewers cannot assess the reliability of the findings.

Item		Recommendation	Section/line number, or reason for not reporting
**Study design**	1	For each experiment, provide brief details of study design including:	
		a. The groups being compared, including control groups. If no control group has been used, the rationale should be stated.	Methods, Animals and treatment section: The sham and HIRI groups were compared (n = 5 per group).
		b. The experimental unit (e.g. a single animal, litter, or cage of animals).	Methods, Animals and treatment section: a single animal.
**Sample size**	2	a. Specify the exact number of experimental units allocated to each group, and the total number in each experiment. Also indicate the total number of animals used.	Methods, Animals and treatment section: n=5 per group, total n=10.
		b. Explain how the sample size was decided. Provide details of any *a priori* sample size calculation, if done.	N/A. Methods, Animal experiments section: Sample size (n = 5 per group) was determined based on previous studies and pilot experiments in our laboratory. No a priori power calculation was performed.
**Inclusion and exclusion criteria**	3	a. Describe any criteria used for including and excluding animals (or experimental units) during the experiment, and data points during the analysis. Specify if these criteria were established *a priori*. If no criteria were set, state this explicitly.	N/A. No specific inclusion or exclusion criteria were applied. All mice that successfully underwent the surgical procedure were included.
		b. For each experimental group, report any animals, experimental units or data points not included in the analysis and explain why. If there were no exclusions, state so.	N/A. Methods, Animal experiments section: No animals were excluded from the analysis.
		c. For each analysis, report the exact value of *n* in each experimental group.	Methods, Animal experiments section: n = 5 per group (sham and HIRI).
**Randomisation**	4	a. State whether randomisation was used to allocate experimental units to control and treatment groups. If done, provide the method used to generate the randomisation sequence.	Methods, Animal experiments section: Randomization was performed using a computer-generated random number sequence.
		b. Describe the strategy used to minimise potential confounders such as the order of treatments and measurements, or animal/cage location. If confounders were not controlled, state this explicitly.	Methods, Animals and treatment section: All the surgeries were performed by the same surgeon under the same experimental conditions to minimize any variations.
**Blinding**	5	Describe who was aware of the group allocation at the different stages of the experiment (during the allocation, the conduct of the experiment, the outcome assessment, and the data analysis).	Methods, Animal experiments section: The surgeon was not blinded to group allocation due to the nature of the surgical procedure. However, all outcome assessments, including histopathological examination, Western blot analysis, and RT?qPCR, were performed by investigators who were blinded to the experimental groups.
**Outcome measures**	6	a. Clearly define all outcome measures assessed (e.g. cell death, molecular markers, or behavioural changes).	Immunofluorescence (global lactylation), lactate accumulation detection, RT-qPCR (NR4A3, PFKFB3, ZYX mRNA), immunohistochemistry (NR4A3, PFKFB3, ZYX protein), Western blot (NR4A3, PFKFB3, ZYX protein).
		b. For hypothesis-testing studies, specify the primary outcome measure, i.e. the outcome measure that was used to determine the sample size.	n level and key LRGs section: The primary outcomes were the expression levels of three key LRGs (NR4A3, PFKFB3, ZYX) at both mRNA and protein levels, and global protein lactylation levels in liver tissues.
**Statistical methods**	7	a. Provide details of the statistical methods used for each analysis, including software used.	y test, comparisons between two groups (sham vs. HIRI) were performed using the unpaired Student's t?test. The level of statistical significance was set at P < 0.05. All statistical analysis was performed using SPSS software.
		b. Describe any methods used to assess whether the data met the assumptions of the statistical approach, and what was done if the assumptions were not met.	Methods, Statistical analysis section: Normality was assessed using the Shapiro–Wilk test; all data passed the normality test, justifying the use of the unpaired Student's t?test.
**Experimental animals**	8	a. Provide species-appropriate details of the animals used, including species, strain and substrain, sex, age or developmental stage, and, if relevant, weight.	Methods, Animals and treatment section: Eight-week-old male C57BL/6J mice, weighing 20–25 g.
		b. Provide further relevant information on the provenance of animals, health/immune status, genetic modification status, genotype, and any previous procedures.	Methods, Animals and treatment section: Obtained from Jiangsu GemPharmatech Company. Specific pathogen-free (SPF) environment. No genetic modifications were involved.
**Experimental procedures**	9	For each experimental group, including controls, describe the procedures in enough detail to allow others to replicate them, including:	
		a. What was done, how it was done and what was used.	went the same surgical procedure, including isolation of the portal vein and hepatic artery, but without vascular occlusion. All surgical procedures were performed by the same surgeon.
		b. When and how often.	inutes, followed by 6 hours of reperfusion. Tissue and serum samples were collected once at the end of the 6-hour reperfusion period. All procedures were performed once per animal.
		c. Where (including detail of any acclimatisation periods).	All animal experiments were conducted at the animal facility of Beijing Friendship Hospital, Capital Medical University. Mice were allowed a one-week acclimatization period before the experiment.
		d. Why (provide rationale for procedures).	to allow sufficient time for tissue injury and lactylation changes to develop. The sham group served as the control to account for the effects of anesthesia and surgical manipulation.
**Results**	10	For each experiment conducted, including independent replications, report:	
		a. Summary/descriptive statistics for each experimental group, with a measure of variability where applicable (e.g. mean and SD, or median and range).	[illegible] D-F). RT-qPCR, immunohistochemistry and Western blot confirmed upregulation of NR4A3, PFKFB3, and ZYX at both mRNA and protein levels in HIRI tissues (P < 0.05, Fig. 10G-I). Data are presented as mean ± SD.
		b. If applicable, the effect size with a confidence interval.	N/A. Effect sizes and confidence intervals were not reported in this study.

The Recommended Set

These items complement the Essential 10 and add important context to the study. Reporting the items in both sets represents best practice.

Item		Recommendation	Section/line number, or reason for not reporting
**Abstract**	11	Provide an accurate summary of the research objectives, animal species, strain and sex, key methods, principal findings, and study conclusions.	[illegible] graft survival in liver transplantation.
**Background**	12	a. Include sufficient scientific background to understand the rationale and context for the study, and explain the experimental approach.	Introduction: The background and scientific rationale for the study are presented.
		b. Explain how the animal species and model used address the scientific objectives and, where appropriate, the relevance to human biology.	Introduction: The mouse HIRI model was selected as a well-established and clinically relevant model for studying hepatic ischemia-reperfusion injury.
**Objectives**	13	Clearly describe the research question, research objectives and, where appropriate, specific hypotheses being tested.	[illegible] validate key LRGs in a mouse HIRI model.
**Ethical statement**	14	Provide the name of the ethical review committee or equivalent that has approved the use of animals in this study, and any relevant licence or protocol numbers (if applicable). If ethical approval was not sought or granted, provide a justification.	Institutional Review Board Statement: The study protocol was approved by the ethics committee of Beijing Friendship Hospital, Capital Medical University, Beijing, China (Approval No. 25-0903, approval date: 24 October 2025). All animal experiments were performed in accordance with the ARRIVE 2.0 guidelines.
**Housing and husbandry**	15	Provide details of housing and husbandry conditions, including any environmental enrichment.	[illegible]ight/dark cycle, and 50 ± 10% humidity).
**Animal care and monitoring**	16	a. Describe any interventions or steps taken in the experimental protocols to reduce pain, suffering and distress.	[illegible] to ensure deep anesthesia throughout the procedure. Animals were housed in a temperature-controlled environment (22 ± 2 °C) with easy access to food and water during recovery. Animals were monitored daily for signs of pain or distress.
		b. Report any expected or unexpected adverse events.	[illegible]ent section: N/A.No unexpected adverse events occurred during the study.
		c. Describe the humane endpoints established for the study, the signs that were monitored and the frequency of monitoring. If the study did not have humane endpoints, state this.	[illegible]ith an overdose of pentobarbital sodium. Animals were monitored daily during the adaptation period and every 6 hours after surgery until tissue collection. No animals reached the humane endpoints during this study.
**Interpretation/ scientific implications**	17	a. Interpret the results, taking into account the study objectives and hypotheses, current theory and other relevant studies in the literature.	Discussion: The findings demonstrate enhanced global lactylation and upregulation of NR4A3, PFKFB3, and ZYX in HIRI tissues, suggesting these LRGs are potential therapeutic targets for mitigating HIRI.
		b. Comment on the study limitations including potential sources of bias, limitations of the animal model, and imprecision associated with the results.	Discussion: These results are discussed in the context of previous studies on lactylation and ischemia-reperfusion injury.
**Generalisability/ translation**	18	Comment on whether, and how, the findings of this study are likely to generalise to other species or experimental conditions, including any relevance to human biology (where appropriate).	Discussion: The findings provide insights into the molecular mechanisms of HIRI and may be generalizable to other ischemia-reperfusion injury models.
**Protocol registration**	19	Provide a statement indicating whether a protocol (including the research question, key design features, and analysis plan) was prepared before the study, and if and where this protocol was registered.	N/A.The experimental protocol was not pre-registered.
**Data access**	20	Provide a statement describing if and where study data are available.	[illegible] be directed to the corresponding authors.
**Declaration of interests**	21	a. Declare any potential conflicts of interest, including financial and non-financial. If none exist, this should be stated.	Conflicts of Interest: The authors declare no conflicts of interest.
		b. List all funding sources (including grant identifier) and the role of the funder(s) in the design, analysis and reporting of the study.	This work was supported by Capital ’ s Funds for Health Improvement and Research (No.2024-1-2022).
