# Supplementary material for: Integration of Single-Cell and Bulk RNA Sequencing Data to Identify Lactylation-Related Gene Signatures in Hepatic Ischemia–Reperfusion Injury Using Machine Learning Algorithms
Source: Int J Mol Sci. 2026 Sep 7;27(17):7965. doi: 10.3390/ijms27177965 (PMC13566055; doi:10.3390/ijms27177965)
Supplement: Supplementary file 1 [file ijms-27-07965-s001.zip › Supplementary Figures S1-S2.pdf]

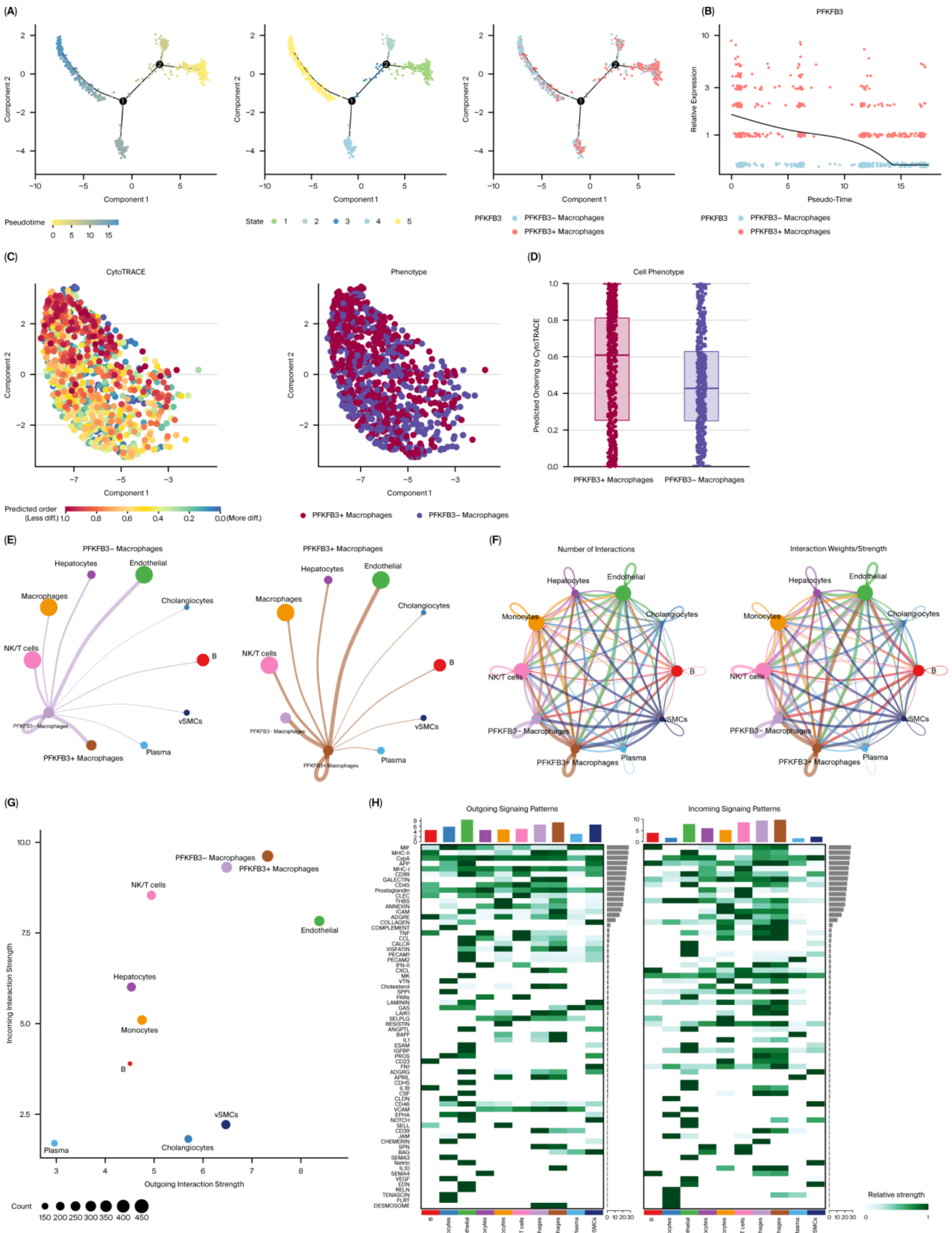
(A)
Component 1
Component 2
Pseudotime
State
PFKFB3
PFKFB3− Macrophages
PFKFB3+ Macrophages
(B)
PFKFB3
Relative Expression
Pseudo-Time
(C)
CytoTRACE
Phenotype
Predicted order
(Less diff.) 1.0
(More diff.)
(D)
Cell Phenotype
Predicted Ordering by CytoTRACE
(E)
Hepatocytes
Endothelial
Macrophages
Cholangiocytes
NK/T cells
B
vSMCs
Plasma
(F)
Number of Interactions
Interaction Weights/Strength
Monocytes
(G)
Incoming Interaction Strength
Outgoing Interaction Strength
Count
(H)
Outgoing Signaling Patterns
Incoming Signaling Patterns
Relative strength

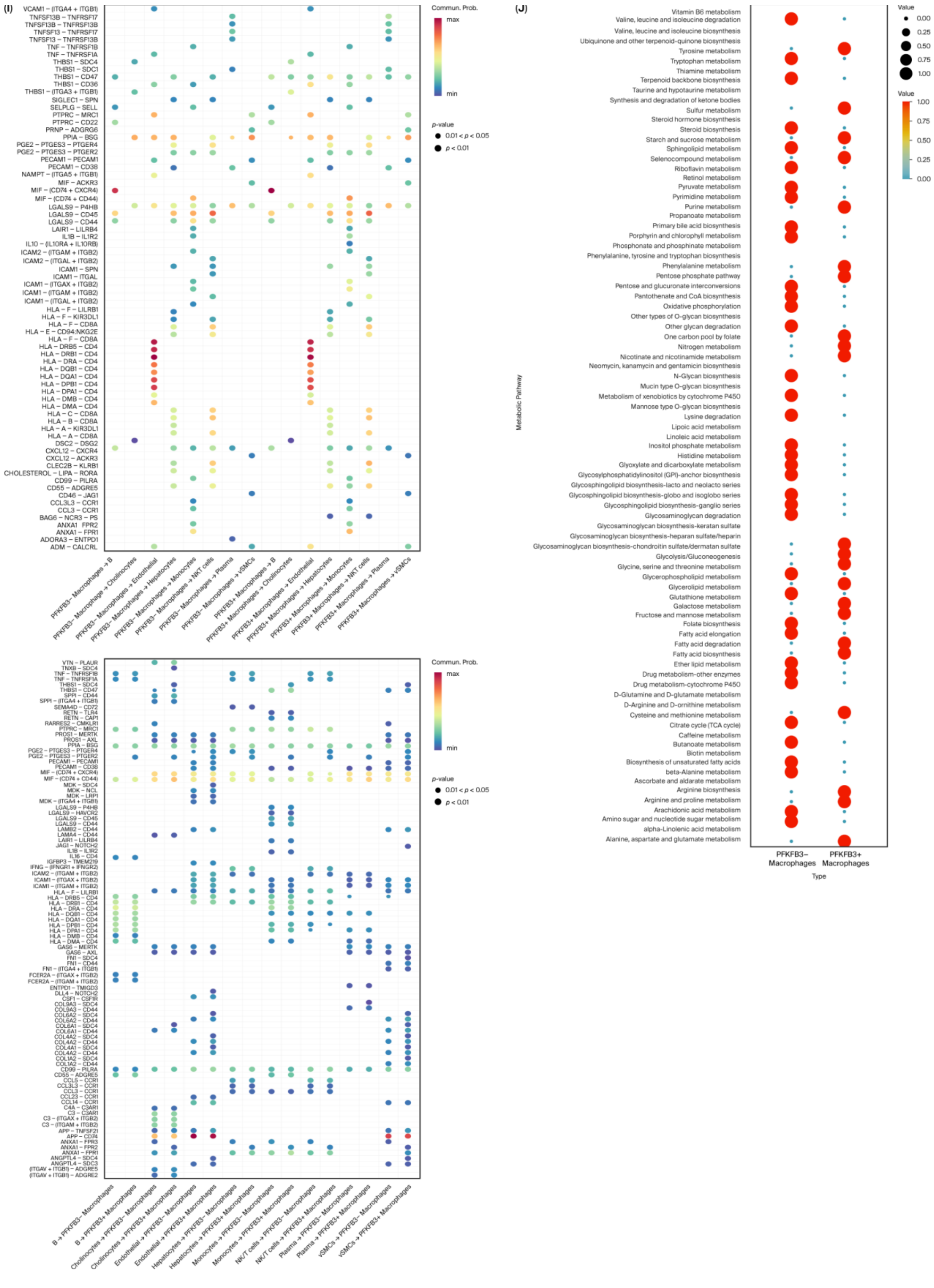

Figure S1 The landscape of cell trajectory and cell-cell communication of PFKFB3. (**A**) Quasi-temporal analysis showing the change of proportions of PFKFB3+macrophages. (**B**) The results of pseudotemporal analysis portrayed the relative expression of PFKFB3. (**C**) CytoTRACE analysis

illustrated cell differentiation level in PFKFB3 labeled macrophages. (**D**) Bar graph showing the differentiation status of PFKFB3 labeled macrophages.(**E-G**) The results of cellular communication indicated the quantity and intensity of cellular communication between PFKFB3+ macrophages and other cell types.(**H**) The heat map depicting the efferent or afferent contributions of all signals to different groups of cells. (**I**) The bubble chart shows ligand–receptor interactions. Bubble size represents p value generated by the permutation test, and the color represents the possibility of interactions.(**J**)The results of metabolic pathway analysis of PFKFB3 in macrophages.

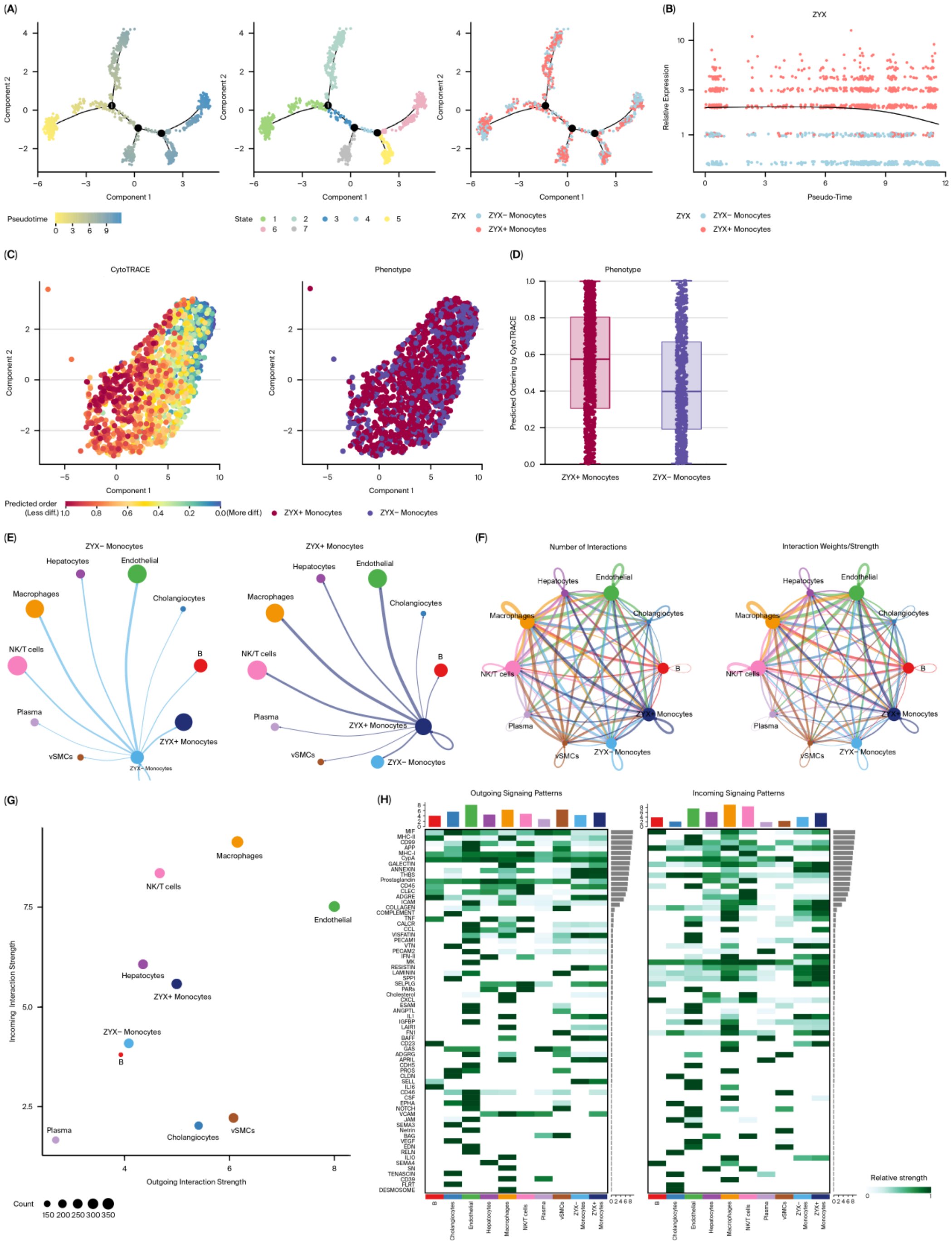

(A)
Component 1
Component 2
Pseudotime
State
ZYX
ZYX− Monocytes
ZYX+ Monocytes
(B)
ZYX
Relative Expression
Pseudo-Time
ZYX− Monocytes
ZYX+ Monocytes
(C)
CytoTRACE
Phenotype
Component 1
Component 2
Predicted order
(Less diff.)
(More diff.)
ZYX+ Monocytes
ZYX− Monocytes
(D)
Phenotype
Predicted Ordering by CytoTRACE
ZYX+ Monocytes
ZYX− Monocytes
(E)
ZYX− Monocytes
ZYX+ Monocytes
Hepatocytes
Endothelial
Macrophages
Cholangiocytes
NK/T cells
B
Plasma
vSMCs
(F)
Number of Interactions
Interaction Weights/Strength
(G)
Incoming Interaction Strength
Outgoing Interaction Strength
Count
150 200 250 300 350
(H)
Outgoing Signaling Patterns
Incoming Signaling Patterns
Relative strength

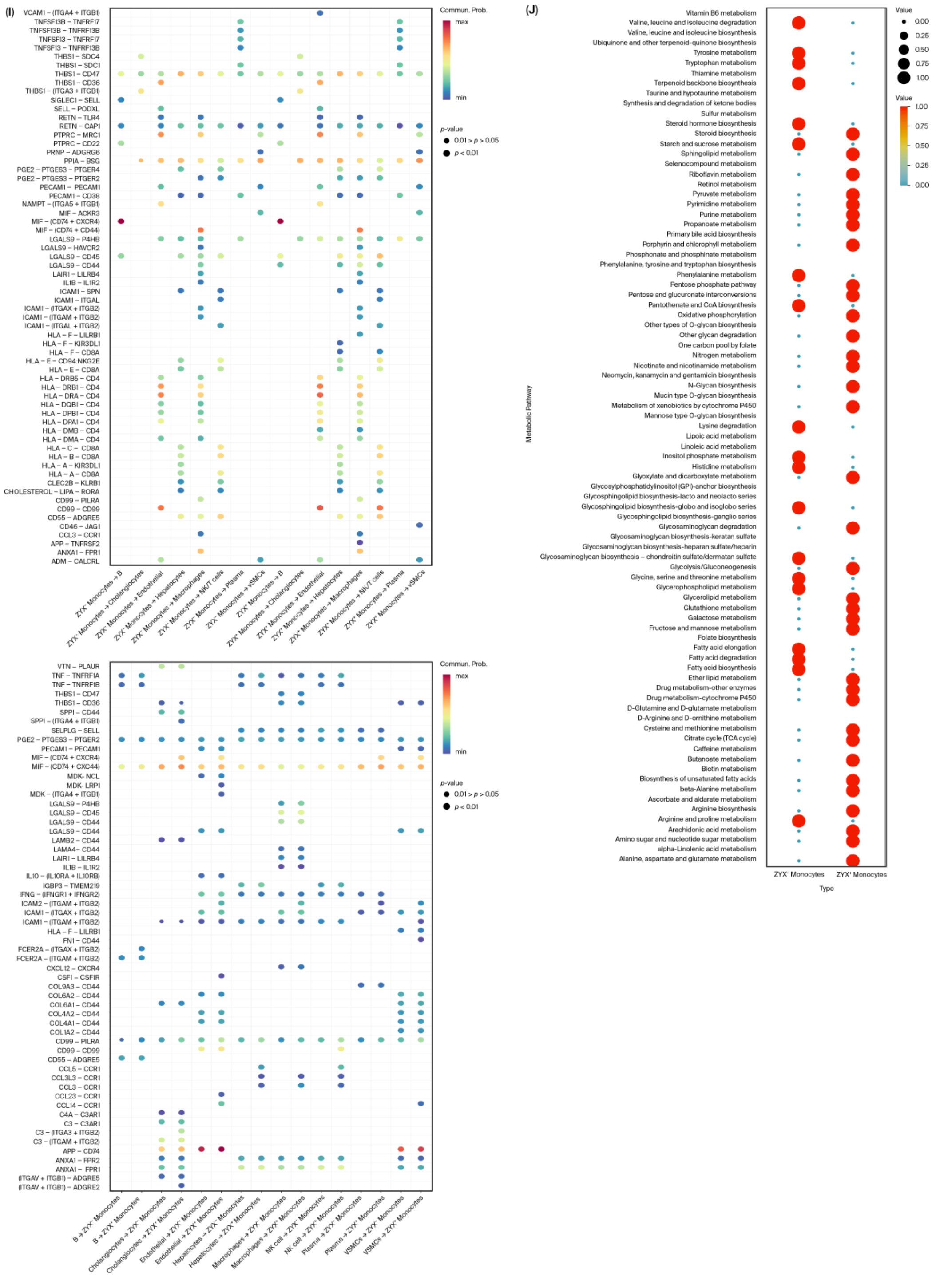

Figure S2 The landscape of cell trajectory and cell-cell communication of ZYX. (**A**) Quasi-temporal analysis showing the change of proportions of ZYX+monocytes. (**B**) The results of pseudotemporal analysis portrayed the relative expression of ZYX. (**C**) CytoTRACE analysis illustrated cell

differentiation level in ZYX labeled monocytes. (**D**) Bar graph showing the differentiation status of ZYX labeled monocytes.(**E-G**) The results of cellular communication indicated the quantity and intensity of cellular communication between ZYX+ monocytes and other cell types.(**H**) The heat map depicting the efferent or afferent contributions of all signals to different groups of cells. (**I**) The bubble chart shows ligand–receptor interactions. Bubble size represents p value generated by the permutation test, and the color represents the possibility of interactions. (**J**) The results of metabolic pathway analysis of ZYX in monocytes.
